# *Caenorhabditis elegans* SWI/SNF Subunits Control Sequential Developmental Stages in the Somatic Gonad

**DOI:** 10.1534/g3.113.009852

**Published:** 2014-01-08

**Authors:** Edward E. Large, Laura D. Mathies

**Affiliations:** * Department of Genetics, North Carolina State University, Raleigh, North Carolina 27695-7614; † Department of Pharmacology and Toxicology, Virginia Commonwealth University, Richmond, Virginia 23298-0613

**Keywords:** SWI/SNF, chromatin remodeling, ehn-3, hnd-1, *C. elegans*

## Abstract

The *Caenorhabditis elegans* somatic gonadal precursors (SGPs) are multipotent progenitors that give rise to all somatic tissues of the adult reproductive system. The *hunchback* and Ikaros-like gene *ehn-3* is expressed specifically in SGPs and is required for their development into differentiated tissues of the somatic gonad. To find novel genes involved in SGP development, we used a weak allele of *ehn-3* as the basis for a reverse genetic screen. Feeding RNAi was used to screen ∼2400 clones consisting of transcription factors, signaling components, and chromatin factors. The screen identified five members of the *C. elegans* SWI/SNF chromatin remodeling complex as genetic enhancers of *ehn-3*. We characterized alleles of 10 SWI/SNF genes and found that SWI/SNF subunits are required for viability and gonadogenesis. Two conserved SWI/SNF complexes, PBAF and BAF, are defined by their unique array of accessory subunits around a common enzymatic core that includes a catalytic Swi2/Snf2–type ATPase. Tissue-specific RNAi experiments suggest that *C. elegans* PBAF and BAF complexes control different processes during somatic gonadal development: PBRM-1, a signature subunit of PBAF, is important for normal SGP development, whereas LET-526, the distinguishing subunit of BAF, is required for development of a differentiated cell type, the distal tip cell (DTC). We found that the SWSN-4 ATPase subunit is required for SGP and DTC development. Finally, we provide evidence that *C. elegans* PBAF subunits and *hnd-1/*dHand are important for the cell fate decision between SGPs and their differentiated sisters, the head mesodermal cells.

One of the fundamental questions in developmental and stem cell biology is how a single progenitor cell can differentiate into multiple cell types, leading to tissue and organ development. The *C. elegans* somatic gonadal precursors (SGPs) are multipotent progenitors that develop into all somatic cells of the adult reproductive system. Two SGPs are generated during embryogenesis and, together with two primordial germ cells (PGCs), comprise the four-celled gonadal primordium (Sulston *et al.* 1983). In hermaphrodites, each SGP generates one of the two U-shape “arms” of the reproductive system via nearly identical and highly stereotyped cell lineages (Kimble and Hirsh 1979). In total, there are 143 cells and five mature tissues in the hermaphrodite somatic gonad, including distal tip cells (DTCs), an anchor cell (AC), sheath, spermatheca, and uterus (Hubbard and Greenstein 2000). We previously identified four transcriptional regulators that are expressed and function early in SGPs: the dHand gene *hnd-1* is required for SGP survival (Mathies *et al.* 2003); the GLI ortholog *tra-1* controls SGP polarity (Mathies *et al.* 2004); and two *hunchback* and Ikaros-like (HIL) genes, *ehn-3* and *R08E3.4/ztf-16*, are required for the development of SGPs into differentiated cell types (Large and Mathies 2010). None of these genes appears to be required for the specification of SGPs and very little is known about the determinants of SGP potency. We used a sensitized RNAi screen to identify additional SGP regulators and found evidence that SWI/SNF chromatin remodeling complexes control early SGP development.

SWI/SNF complexes are large, multi-subunit complexes that utilize the energy of ATP hydrolysis to alter the interaction between nucleosomes and DNA, thereby affecting the accessibility of DNA and influencing gene expression (Clapier and Cairns 2009). In humans, the complexes are approximately 2 mDa in size, contain 8–11 subunits, and are encoded by at least 21 genes (Hargreaves and Crabtree 2011; Ho *et al.* 2009; Wu 2012). The overall subunit composition of the complexes is conserved across phyla; however, individual subunits are often encoded by more than one gene in mammals (Figure 1A). The enzymatic core consists of four subunits that have full chromatin remodeling activity *in vitro* (Phelan *et al.* 1999). Central to this activity is a Swi2/Snf2-type ATPase, BRM or BRG1 in mammals and Brahma in *Drosophila*. Mutation of the core subunits results in early lethality in mice and flies (Brizuela *et al.* 1994; Bultman *et al.* 2000; Crosby *et al.* 1999; Dingwall *et al.* 1995; Guidi *et al.* 2001; Kim *et al.* 2001; Klochendler-Yeivin *et al.* 2000; Roberts *et al.* 2000; Tamkun *et al.* 1992), underscoring the importance of SWI/SNF for development.

**Figure 1:**
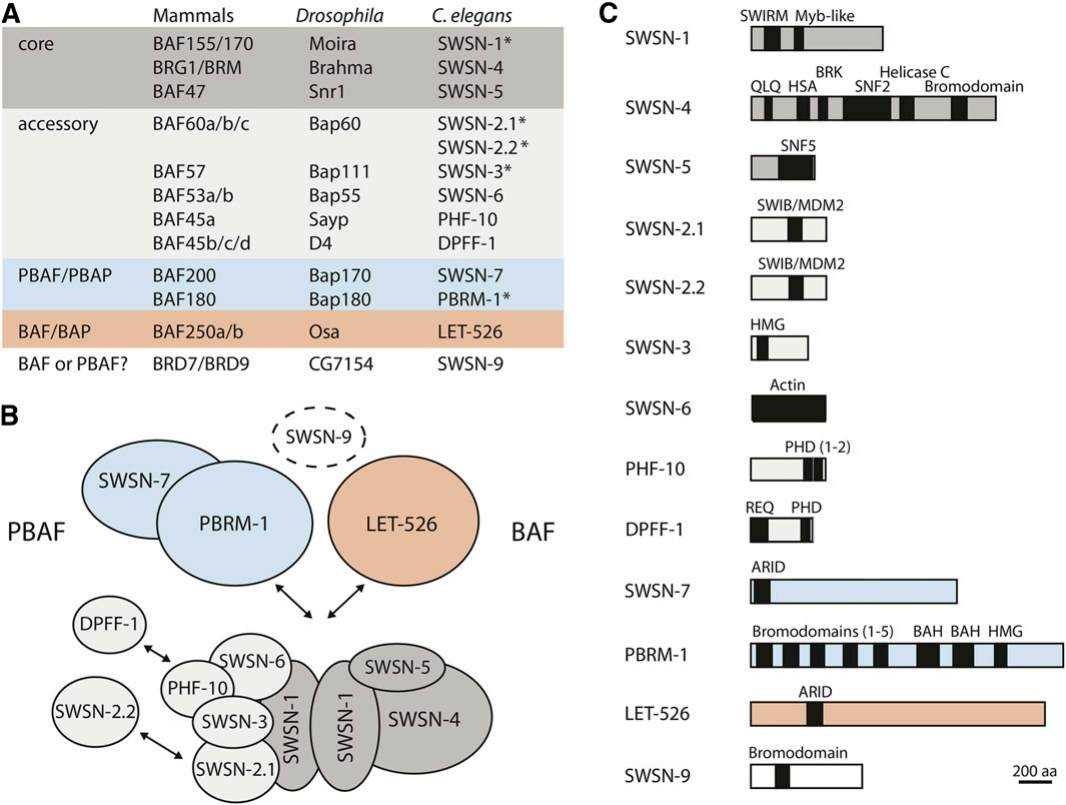
*C*. *elegans* SWI/SNF chromatin remodeling complexes. The *C. elegans* genome contains homologs of all SWI/SNF subunits. We classified them as core (dark gray), accessory (light gray), or complex-specific (PBAF, blue; BAF, orange) based on the purified mammalian complexes. SWSN-9 is considered a potential BAF or PBAF subunit because of its similarity to BRD7 and BRD9, which are subunits of PBAF and BAF, respectively. (A) Mammalian, *Drosophila*, and *C. elegans* proteins are listed. Subunits identified in our *ehn-3* enhancer screen are indicated with asterisks (*). (B) By analogy with mammalian SWI/SNF, the *C. elegans* subunits are predicted to combine to form molecularly and functionally distinct complexes. (C) *C. elegans* SWI/SNF proteins, including all major domains. Only the longest isoform is listed.

In addition to the enzymatic core, SWI/SNF complexes contain common and complex-specific accessory subunits (Figure 1A). Two major subfamilies of SWI/SNF, called Brg/Brm–associated factors (BAF) and Polybromo-BAF (PBAF), are distinguished by their unique accessory subunits. The homologous complexes in *Drosophila* are called BAP and PBAP. BAF/BAP complexes contain the ARID protein, BAF250a or BAF250b in mammals and Osa in *Drosophila*. PBAF/PBAP complexes contain two well-characterized subunits: the bromodomain protein BAF180 in mammals and Bap180/Polybromo in *Drosophila* and the ARID protein BAF200 in mammals and Bap170 in *Drosophila* (Chalkley *et al.* 2008; Kwon and Wagner 2007; Mohrmann and Verrijzer 2005). BAF/BAP and PBAF/PBAP have overlapping and distinct functions in mammalian gene regulation and *Drosophila* development (Gao *et al.* 2008; Huang *et al.* 2008; Lemon *et al.* 2001; Moshkin *et al.* 2007; Wang *et al.* 2004; Yan *et al.* 2005; Yan *et al.* 2008). These functional differences likely result from targeting of the two complexes to different chromosomal regions, as has been demonstrated in *Drosophila* (Mohrmann *et al.* 2004).

The *in vivo* roles of common accessory subunits are less well-characterized, but evidence to date suggests that they are important for the functional specificity of the complexes. For example, mammalian neural progenitors utilize a complex called npBAF that contains BAF45a and BAF53a, whereas differentiated neurons use a complex called nBAF that contains BAF45b and BAF53b (Lessard *et al.* 2007). The switch from npBAF to nBAF is essential for the cessation of proliferation and the differentiation of neuronal subtypes. Furthermore, BAF53a cannot substitute for BAF53b in neurons (Wu *et al.* 2007), suggesting that this accessory protein provides biological specificity to the complex. Therefore, specific combinations of accessory subunits play a key role in determining the functional diversity of SWI/SNF complexes.

*C. elegans* SWI/SNF genes were initially identified for their role in the asymmetric division of a tail hypodermal cell, the T cell (Sawa *et al.* 2000). They have since been identified as genetic regulators of larval development (Cui *et al.* 2004), the UV DNA damage response (Lans *et al.* 2010), hermaphrodite-specific neuron (HSN) development (Weinberg *et al.* 2013), and gonadogenesis (Shibata *et al.* 2012). More recently, proteomics approaches identified SWI/SNF subunits in association with two transcription factors, DAF-16 and SOMI-1 (Hayes *et al.* 2011; Riedel *et al.* 2013). Genetic, developmental, and proteomic analyses of predicted SWI/SNF subunits are beginning to reveal key similarities and differences between *C. elegans* SWI/SNF complexes and their mammalian counterparts.

Here, we characterized alleles of several *C. elegans* SWI/SNF subunits and found that they are broadly required for viability and act in parallel to *ehn-3* in the somatic gonad. We used tissue-specific RNAi to examine the functions of predicted BAF and PBAF subunits and showed that they control distinct events during gonadogenesis. Finally, we found that genes encoding PBAF subunits have a mutant phenotype that is strikingly similar to that of *hnd-1* mutants and provide evidence that *hnd-1* and PBAF are important for distinguishing multipotent SGPs from their differentiated sisters.

## Materials and Methods

### Strains

*C. elegans* strains were cultured as described previously (Brenner 1974; Wood 1988). All strains were grown at 20° unless otherwise specified and were derived from the Bristol strain N2. The following mutations were used in this study and are described in *C. elegans II* (Hodgkin 1997), cited references, or the following work:

*LGI:* *pbrm-1**(tm415)* (Shibata *et al.* 2012), *pbrm-1**(ok843)*, *let-526(*h185*)* (Johnsen *et al.* 2000), *let-526(gk816)*, *let-526**(tm4795)*, *swsn-2.2(ok3161)* (Weinberg *et al.* 2013), *swsn-2.2**(tm3395)*, *swsn-9(ok1354)*

*LGII: swsn-7(gk1041)*, *swsn-7(tm4263)*, *rrf-3(pk1426)*

*LGIII:* ham-3/*swsn-2.1**(tm3309)* (Weinberg *et al.* 2013), *snfc-5*/*swsn-5**(**ok622**)* (Lans *et al.* 2010), **swsn-3*(*tm3647*)*

*LGIV:* *ehn-3**(**q766**)* (Mathies *et al.* 2004), *ehn-3**(**rd2**)* (Large and Mathies 2010), *psa-4*/*swsn-4**(**os13**)* (Sawa *et al.* 2000), *psa-4*/*swsn-4**(**tm305**)*

*LGV:* *psa-1*/*swsn-1**(**ku355**)* (Cui *et al.* 2004), *psa-1*/*swsn-1**(**os22**)* (Sawa *et al.* 2000), **psa-1**/**swsn-1***(**tm4567**)*, *rde-1**(**ne219**)* (Tabara *et al.* 1999), *rol-9**(**sc148**)*

*LGX*: *hnd-1(q740)* (Mathies *et al.* 2003), *ztf-16(tm2127)* (Large and Mathies 2010)

The following balancer chromosomes and molecular markers were used in this study: *hT2**[bli-4(e937) let-?(q782) qIs48]* for LGI and LGIII (referred to as *hT2g*), *qC1**[dpy-19(e1259) glp-1(q339) qIs26]* and *sC1**[dpy-1(s2170)]* for LGIII, *mIn1**[dpy-10(e128) mIs14]* for LGII, and *nT1**[let-? qIs51]* (referred to as *nT1g*) for LGIV and LGV, rdIs2 *[ehn-3A::GFP]* (Welchman *et al.* 2007), *rdIs11* [*ehn-3A::tdTomato]*, *rdIs4* [*ehn-3A::Venus]*, *ccIs4444* *[arg-1::GFP]* (Kostas and Fire 2002).

### Regulome RNAi screen

We compiled a set of ∼2400 RNAi clones consisting of transcription factors, cell signaling components, and chromatin factors from two commercially available genome-wide RNAi libraries, the *C. elegans* ORF-RNAi library (Source BioScience, Nottingham, UK) (Rual *et al.* 2004) and the *C. elegans* RNAi library (Source BioScience, Nottingham, UK) (Kamath *et al.* 2003). Our “regulome” sublibrary included the published signal transduction, transcriptional regulation, and chromatin remodeling libraries (Reece-Hoyes *et al.* 2005), chromatin factors described in WormBook (Cui and Han 2007), and the Ahringer chromatin, phosphatase, and transcription factor libraries (Source BioScience, Nottingham, UK). Feeding RNAi was performed essentially as reported by Kamath *et al.* (2003). Briefly, L4 worms fed on RNAi bacteria for 24 hr, were transferred in duplicate to new wells seeded with RNAi bacteria, and gravid adult worms were allowed to lay eggs for a 24-hr period. The *ehn-3(q766)* worms were screened in parallel to N2 in adjacent columns of a 12-well plate. Defects in gonadal morphology were assessed in the F1 generation using a dissecting microscope. At least 50 animals were examined per well and the penetrance was recorded. We scored as positive any clones that resulted in more than 10% gonadogenesis defects in either the wild-type or *ehn-3(q766)* background. All RNAi clones that gave similar results in a secondary screen were sequenced to verify the clone identity.

### Identification and classification of *C. elegans* SWI/SNF genes

Potential *C. elegans* SWI/SNF genes were identified using a combination of BLAST searches, OrthoList, and previously published articles (Altschul *et al.* 1997, 2005; Cui *et al.* 2004; Sawa *et al.* 2000; Shaye and Greenwald 2011; Shibata *et al.* 2012; Wu *et al.* 2009; Yoo and Crabtree 2009). For subunits with more than one *C. elegans* homolog, we used sequence alignments and protein domain architecture to identify the most likely ortholog(s). Sequences were aligned using Clustal Omega (Sievers *et al.* 2011) in Jalview 2.8 (Waterhouse *et al.* 2009). The results are summarized in Suppporting Information, File S1. Two *C. elegans* genes, *psa-4/swsn-4* and *C52B9.8*, encode proteins with significant sequence similarity to human BRM and BRG1. SWSN-4 had been previously described as a *C. elegans* SWI/SNF subunit (Cui *et al.* 2004; Sawa *et al.* 2000). SWSN-4 contains all seven domains found in human BRM/BRG1, whereas C52B9.8 contains only the HSA, SNF2, and Helicase C domains (File S1). RNAi knockdown of *C52B9.8* did not affect viability or gonadogenesis (data not shown); whether or not *C52B9.8* retains overlapping functions with *swsn-4* remains to be determined. Two genes, *swsn-2.1/ham-3* and *swsn-2.2*, encode proteins with sequence similarity to mammalian BAF60 proteins. The *C. elegans* proteins are not clear orthologs of any specific BAF60 protein; instead, the worm and mammalian genes appear to have duplicated independently (Weinberg *et al.* 2013). Two genes, *phf-10* and *dpff-1*, encode proteins with sequence similarity to mammalian BAF45 proteins. *C. elegans* PHF-10 is most closely related to BAF45a/PHF10, whereas DPFF-1 is equally similar to BAF45b, c, and d (also known as DPF1-3). The DPF proteins all contain an N-terminal Requiem domain that is not present in PHF10 (File S1). RNAi knockdown of *phf-10* resulted in gonadogenesis defects that were stronger in the *ehn-3(q766)* background, whereas RNAi of *dpff-1* did not affect viability or gonadogenesis (data not shown). We classified PHF-10 and DPFF-1 as common accessory subunits in accordance with previous publications; however there is some evidence to suggest that human BAF45a/PHF10 may be predominantly found in PBAF complexes, whereas BAF45b/c/d (DPF1-3) are found in BAF complexes (Middeljans *et al.* 2012). Finally, *C. elegans* has a single protein, SWSN-9, with similarity to mammalian BRD7 and BRD9. Human BRD7 and BRD9 have 27.1% and 23.0% amino acid identity with SWSN-9, respectively, compared to 36.7% shared amino acid identity between the human proteins. To clarify the relationship between SWSN-9 and the mammalian proteins, we constructed a phylogenetic tree using maximum likelihood and maximum parsimony analysis in Mega 5.2.2 (Tamura *et al.* 2011). BRD7/9 genes were identified using BLAST searches with the human BRD7 and BRD9 proteins (File S1). *C. elegans* SWSN-9 is not an obvious ortholog of mammalian BRD7 or BRD9; instead, the ancestral BRD7/9 protein appears to have duplicated in vertebrates (Figure S3).

### Genetic and molecular analysis of SWI/SNF alleles

SWI/SNF alleles used in this study are listed in Table 1. Deletion alleles obtained from the International *C. elegans* Gene Knockout Consortium or National BioResource Project were crossed alternately to N2 and an appropriate balancer chromosome at least three times (a total of six outcrosses), and the breakpoint was sequenced to verify the molecular lesion (Table S1 and Figure S1). Complementation tests were performed for genes with more than one allele: *swsn-1(tm4567)* failed to complement *swsn-1(os22)* at 25°; *swsn-2.2(tm3395)* failed to complement *swsn-2.2(ok3161)*; *swsn-4(tm305)* failed to complement *swsn-4(os13)* at 25°; *swsn-7(gk1041)* failed to complement *swsn-7(tm4263)*; *pbrm-1(ok843)* failed to complement *pbrm-1(tm415)*; and *let-526(tm4795)* and *let-526(gk816)* failed to complement *let-526(h185)*. These results suggest that the observed phenotypes are attributable to the deletions and not to linked mutations.

**Table 1:** Alleles used in this study

Gene	Allele	Mutation	Affect on Locus	Potential Null
*swsn-1/psa-1*	*os22*	Missense	P86S	No
	*ku355*	Missense	P86L	No
	*tm4567*	Insertion/deletion	Frame shift after E60	Yes
*swsn-2.1*	*tm3309*	Insertion/deletion	Frame shift after V67	Yes
*swsn-2.2*	*tm3395*	Deletion	Frame shift after K65	Yes
	*ok3161*	Deletion	Frame shift after I62	Yes
*swsn-3*	*tm3647*	Insertion/deletion	Promoter/start deletion	No*^a^*
*swsn-4/psa-4*	*os13*	Missense	T673I L697F	No
	*tm305*	Deletion	Frame shift after G630	Yes
*swsn-5*	*ok622*	Deletion/duplication	Unknown	No*^a^*
*swsn-7*	*gk1041*	Deletion	Frame shift after A189	Yes
	*tm4263*	Deletion	Deletion of R40 – W406	Yes
*swsn-9*	*ok1354*	Deletion	Frame shift after I197	Yes
*pbrm-1*	*ok843*	Deletion	Frame shift after I390	Yes*^b^*
	*tm415*	Insertion/deletion	Frame shift after L539	Yes*^b^*
*let-526*	*h185*	Nonsense	Q473stop	Yes
	*gk816*	Insertion/deletion	Frame shift after R47	Yes
	*tm4795*	Deletion	Frame shift after A1248	Yes

a See Figure S2.

b Deletion affects *pbrm-1a*; *pbrm-1b* may be unaffected.

The *swsn-5(ok622)* and *swsn-3(tm3647)* deletion alleles did not display phenotypes observed in previous RNAi experiments (Kamath *et al.* 2003; Rual *et al.* 2004; Sawa *et al.* 2000); therefore, we outcrossed the strains further and analyzed them molecularly. PCR from genomic DNA or single-stranded cDNA was used to detect duplication events or transcripts corresponding to the locus, respectively. For RT-PCR, mixed stage worms were washed from nearly starved plates and RNA was prepared using TRIzol (Invitrogen). First strand cDNA was synthesized using Superscript II (Invitrogen) and oligo dT. For genomic PCR, mixed stage worms were treated with proteinase K and used directly as template. PCR assays failed to detect an additional copy of *swsn-3* in the *swsn-3(tm3647)* deletion strain. However, RT-PCR indicated that the 3′ end of the locus was expressed in *swsn-3(tm3647)* homozygotes (Figure S2); therefore, *tm3647* may retain some activity. PCR and RT-PCR revealed that the *swsn-5* coding region is present and expressed in *swsn-5(ok622)* homozygotes (Figure S2), indicating that this strain contains a secondary duplication of the locus.

### Phenotypic analysis

Three to 12 (depending on viability) young adults were placed on each of three plates and allowed to lay eggs for 24 hr. Gonadogenesis defects were assessed in the F1 generation at the fourth larval stage using a dissecting microscope; the number of worms with missing gonadal arms, disorganized gonads, or no visible gonad were recorded. The average penetrance and SD of these gonadogenesis defects were calculated. The frequency of missing anterior gonadal arms was calculated relative to the number of worms with one missing arm. Unpaired *t*-tests were used for pairwise statistical comparisons and the raw p-value is reported.

### Tissue-restricted RNAi

The mesodermal RNAi strain was generated by cloning *rde-1* genomic sequence, including all exons, introns, and UTRs, downstream of the *hnd-1* promoter from pJK848 (Mathies *et al.* 2003). The resulting plasmid, pRA279, was injected into *rrf-3(pk1426)*; *unc-119(ed3)*; *rde-1(ne219)* with an *unc-119* rescue construct as a co-injection marker (Maduro and Pilgrim 1995) and integrated with gamma irradiation to create *rdIs7[hnd-1*::*rde-1]*. The strain was backcrossed to *unc-119(ed3)*; *rde-1(ne219)* and the *rrf-3* mutation was removed. RNAi was performed by injection of double-stranded RNA (dsRNA) into the gonad. dsRNA was synthesized using the Megascript T7 kit (Ambion) and injected at 0.2–1 mg/ml. In each case, the template contained at least 500 bp of coding sequence. Available RNAi clones were used as template for *swsn-1*, *pbrm-1*, and *let-526* (Kamath *et al.* 2003; Rual *et al.* 2004). The *swsn-4* RNAi clone was generated by amplifying 1680 bp of *swsn-4* genomic sequence with primers RA714/RA715 (Table S2) using a two-step PCR protocol to add T7 promoter sequence and restriction sites for cloning (Dudley and Goldstein 2005).

### Reporter constructs

*ehn-3A*::*Venus* (pRA264) was created by amplifying the *ehn-3A* promoter and 5′UTR from pRA230 using primers RA291/RA314 (Table S2) and cloning the *Xba*I to *Bam*HI fragment into the pPD95.77 expression vector containing the Venus variant of GFP. This construct was then truncated at a BglII site, retaining 948 bp upstream of the *ehn-3A* ATG. *ehn-3A*::*tdTomato* (pRA351) was generated by cloning the *Bgl*II to *Bam*HI fragment of pRA230 into the pPD95.77 expression vector containing tdTomato; this construct retains 948 bp upstream of the *ehn-3A* ATG to the *Pst*I site in the second *ehn-3A* exon. pRA255 and pRA351 were injected into *unc-119(ed3)*; *him-5(e1490)* with an *unc-119* rescue construct as a co-injection marker (Maduro and Pilgrim 1995) and integrated by gamma irradiation to create *rdIs4* [*ehn-3A*::*Venus*] and *rdIs11* [*ehn-3A*::*tdTomato*]. Reporters were visualized using a Zeiss Axioskop II or Zeiss LSM710 microscope.

## Results

The reproductive system develops from a four-celled primordium containing two SGPs and two PGCs. SGPs are generated during embryogenesis from mesodermal lineages (Sulston *et al.* 1983) and remain quiescent until the larval stages, when they undergo several rounds of cell division to generate all 143 cells of the adult hermaphrodite gonad (Kimble and Hirsh 1979). Of particular importance are two DTCs, which are critical for generating the proper morphology and maintaining the function of the reproductive system. DTCs guide the elongation of the two gonadal “arms” to produce the U-shape hermaphrodite gonad and they provide a signal to the adjacent germ cells to promote their mitotic proliferation (Kimble and White 1981). The *C. elegans hunchback* and Ikaros-like gene, *ehn-3*, is expressed specifically in SGPs and is important for the development of several differentiated tissues of the somatic gonad, including DTCs (Large and Mathies 2010; Mathies *et al.* 2004). Despite the importance of *ehn-3* for gonadogenesis, it is not known how this zinc finger transcription factor acts in SGPs to control their subsequent development.

To learn more about how *ehn-3* controls SGP development and to identify new SGP regulators, we used a weak allele of *ehn-3* to provide a sensitized background for an RNAi screen. *ehn-3(q766)* mutants have a low penetrance of missing gonadal arms and this phenotype is strongly enhanced by reducing the function of other SGP regulators, such as *hnd-1* and *tra-1* (Mathies *et al.* 2003, 2004). We limited our screen to genes involved in transcriptional regulation, including transcription factors, signaling components, and chromatin factors (see Materials and Methods). We used feeding RNAi and we screened L4 larvae for gross defects in gonadal morphology using a dissecting microscope. Positives from the primary screen were retested and the clones were sequenced to verify their identity. From a screen of ∼2400 genes, we identified 33 *ehn-3* enhancers (File S2). Among these were five members of the *C. elegans* SWI/SNF chromatin remodeling complex. This result was intriguing in light of the functional similarities between *ehn-3* and Ikaros (Large and Mathies 2010) and the physical interaction between Ikaros and SWI/SNF in mammalian lymphocytes (Kim *et al.* 1999; O’Neill *et al.* 2000). Therefore, we focused on defining the role(s) of SWI/SNF complexes in somatic gonad development.

### *C. elegans* SWI/SNF

The *C. elegans* genome encodes homologs of all mammalian SWI/SNF subunits (see Materials and Methods) (Figure 1). SWI/SNF complexes have not been biochemically purified in *C. elegans*; therefore, we used the mammalian complexes as a framework for our genetic analysis (Figure 1B) (Ho *et al.* 2009; Wang *et al.* 1996a, 1996b). Accordingly, we expect that distinct BAF and PBAF complexes will be defined by their unique accessory subunits, with BAF containing LET-526/BAF250 and PBAF containing PBRM-1/BAF180 and SWSN-7/BAF200. SWSN-9 is the single *C. elegans* homolog of mammalian BRD7 and BRD9 (Figure S3). Because BRD7 is found in PBAF, whereas BRD9 is found in BAF (Kadoch *et al.* 2013; Kaeser *et al.* 2008; Middeljans *et al.* 2012), we included SWSN-9 as a potential BAF or PBAF subunit. Finally, we considered that functional diversity might result from the utilization of different paralogs of the accessory proteins, BAF60 (SWSN-2.1 and SWSN-2.2) and BAF45 (PHF-10 and DPFF-1), as has been proposed for mammalian BAF complexes (Ho and Crabtree 2010). Alleles exist for many of the *C. elegans* SWI/SNF genes (Table 1); however, only a few have been characterized genetically (Cui *et al.* 2004; Lans *et al.* 2010; Sawa *et al.* 2000; Shibata *et al.* 2012; Weinberg *et al.* 2013).

Consistent with a broad role in development, we found that many of the SWI/SNF subunits are required for embryonic or larval viability. We began with deletion alleles of the core genes, *swsn-1*, *swsn-4*, and *swsn-5*. We found that *swsn-1(tm4567)* homozygotes were larval lethal and *swsn-4(tm305)* homozygotes were embryonic lethal (Table 2). Temperature-sensitive alleles of *swsn-1* and *swsn-4* are embryonic lethal at the restrictive temperature (Cui *et al.* 2004; Sawa *et al.* 2000); therefore, our results indicate that *swsn-1* has a maternal contribution that is necessary for embryogenesis. Unlike *swsn-1* and *swsn-4* deletions, the *swsn-5(ok622)* deletion was homozygous viable and displayed no maternal effect lethality (*n* = 1125). This deletion was hypersensitive to UV damage, as were temperature-sensitive alleles of *swsn-1* and *swsn-4* (Lans *et al.* 2010). However, our molecular analysis showed evidence for a secondary duplication of the locus in *swsn-5(ok622)* homozygotes (Figure S2). Because *swsn-5* RNAi results in embryonic lethality (E. E. Large and L. D. Mathies, unpublished observations; Sawa *et al.* 2000), we conclude that *ok622* is not a strong loss-of-function allele. We also examined alleles of the accessory genes, *swsn-2.1*, *swsn-2.*2, and *swsn-3*. Deletion alleles of *swsn-2.1* or *swsn-2.2* were homozygous viable, but they exhibited highly penetrant maternal effect lethality (Table 2). A recent article described distinct functions for *swsn-2.1* and *swsn-2.2* in the hermaphrodite specific neurons (Weinberg *et al.* 2013). To determine whether these genes retain overlapping functions, we generated a balanced strain containing *swsn-2.1(tm3309)* and *swsn-2.2(ok3161)*. Double mutants derived from this strain did not survive beyond the L2 larval stage, indicating that *swsn-2.1* and *swsn-2.2* are redundant for embryonic or early larval development. Finally, we found that a *swsn-3* deletion allele was homozygous viable and displayed no maternal effect lethality (*n* = 576). Molecular analysis indicates that *swsn-3(tm3647)* contains mRNA that could produce a protein lacking the HMG domain but retaining the conserved NHRLI domain (Figure S2). Experiments with the *Drosophila* homolog of *swsn-3*, Bap111, indicate that sequences outside of the HMG domain may be important for its function (Papoulas *et al.* 2001). Therefore, it is likely that *swsn-3(tm3647)* retains some gene activity. In summary, our genetic analysis implicates SWI/SNF core (*swsn-4* and *swsn-1*) and accessory (*swsn-2.1* and *swsn-2.2*) subunits in embryonic or larval development.

**Table 2:** Embryonic and larval lethality of SWI/SNF alleles

Parental Genotype*^a^*	Embryonic Lethality %*^b^*	Larval Lethality %*^b^*	*n*
*swsn-1(tm4567)/*+	2.5	26.8	276
*swsn-2.1(tm3309)*	41.3	24.2	256
*swsn-2.2(tm3395)*	77.5	19.7	806
*swsn-2.2(ok3161)*	85.9	10.7	347
*swsn-4(tm305)/*+	28.9	2.2	277
*swsn-7(tm4263)*	100	0	454
*swsn-7(gk1041)*	100	0	189
*swsn-9(ok1354)*	6.5	42.7	651
*pbrm-1(ok843)*	2.6	96.0	623
*pbrm-1(tm415)*	1.3	20.9	949
*let-526(gk816)/*+	0.8	26.6	237

a Alleles were backcrossed before analysis; however, linked mutations may still be present.

b Embryonic and larval lethality were assessed using a dissecting microscope and are reported as a percentage of the total progeny (*n*).

Next, we examined alleles of predicted BAF and PBAF genes (Figure 1A). We found that *let-526(gk816)* and *let-526(tm4795)* homozygotes died as early larvae (Table 2; data not shown), similar to previously characterized alleles of *let-526* (Shibata *et al.* 2012). Deletion alleles of *pbrm-1* are homozygous viable and resulted in maternal effect larval lethality (Table 2). The two *pbrm-1* alleles differ in their phenotypic severity: *pbrm-1(ok843)* was mostly larval lethal, whereas *pbrm-1(tm415)* had a higher percentage of viable progeny. The *pbrm-1(ok843)* and *pbrm-1(tm415)* deletion alleles express mRNAs encoding proteins that are truncated after the second or third bromodomains, respectively. Therefore, *pbrm-1(ok843)* is a stronger loss-of-function allele by molecular and genetic criteria. Both alleles of *swsn-7* were homozygous viable and resulted in 100% maternal effect embryonic lethality (Table 2). Therefore, two PBAF genes, *swsn-7* and *pbrm-1*, have different terminal phenotypes. Finally, we examined the function of *swsn-9*, which is a potential BAF or PBAF gene. The *swsn-9(ok1354)* deletion allele was viable and resulted in partial maternal effect larval lethality, similar to *pbrm-1* alleles. We conclude that BAF and PBAF genes are required for viability and specific subunits appear to play distinct roles in early development.

**Figure 2:**
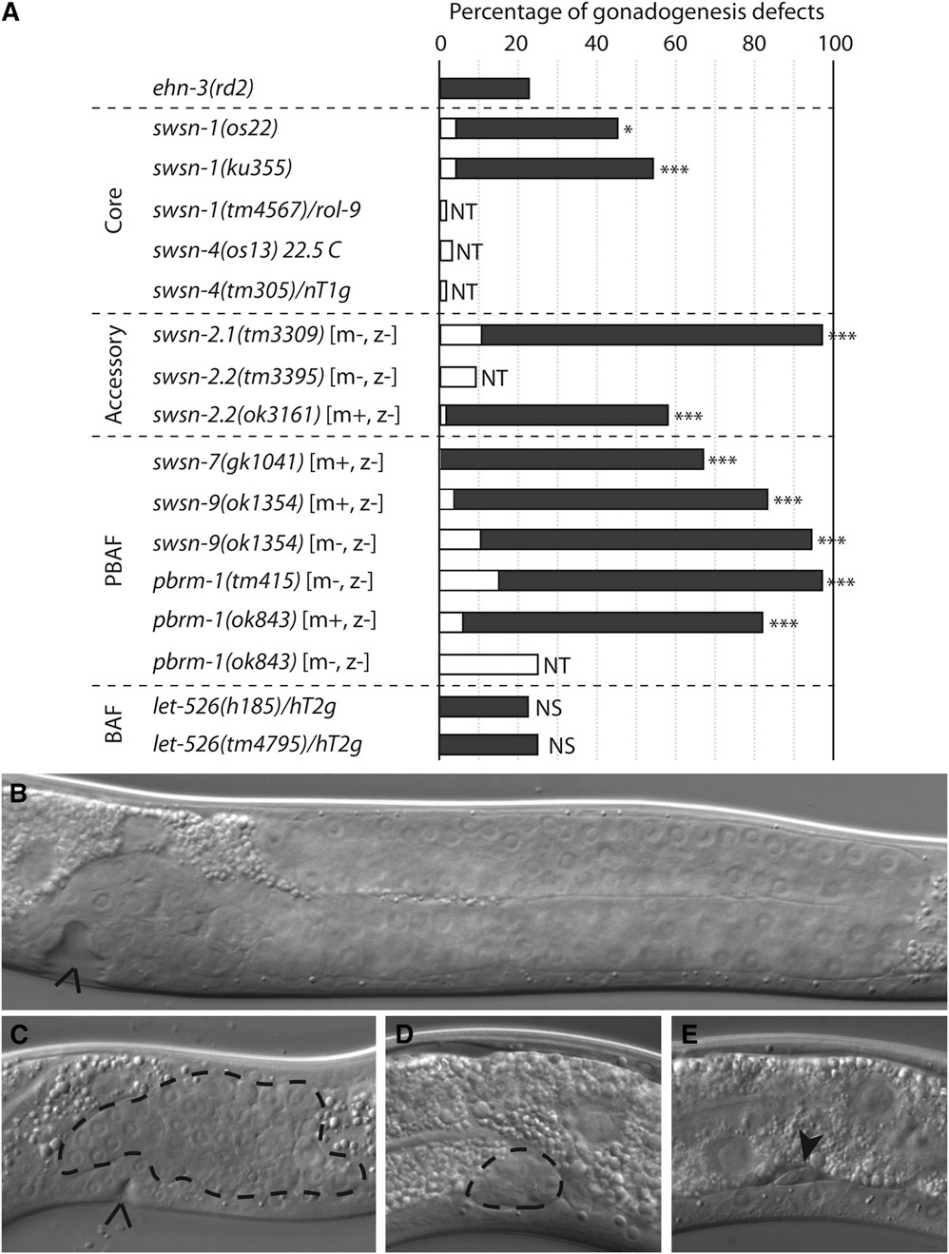
*C*. *elegans* subunits act alone and in parallel to *ehn-3* in the somatic gonad. (A) SWI/SNF mutants have gonadogenesis defects alone or in combination with *ehn-3(rd2)*. Genotype is indicated on the left; maternal [m+ or m−] and zygotic [z+ or z−] contributions are indicated when relevant. Percentages of animals with gonadogenesis defects in an otherwise wild-type (white bars) or *ehn-3(rd2)* background (black bars) are shown. Unpaired *t*-tests were used for statistical comparisons; *ehn-3(rd2)* was compared and the significance is reported (NS, not significant; *P ≤0.05; ***P ≤ 0.001). NT indicates double mutant combinations that were not tested. Additional data are found in Table S3. (B–E) DIC images showing the range of gonadogenesis defects seen in *pbrm-1(ok843)*; *ehn-3(rd2)* double mutants. (B) Missing anterior gonadal arm (posterior is seen), (C) unextended gonad with a clear patch of gonadal tissue, or (D, E) severely reduced gonad. Dashed lines and arrowheads indicate gonadal tissue; carats point to the vulva when present.

Finally, we investigated the requirement for SWI/SNF in the somatic gonad. To do this, we examined viable SWI/SNF alleles for defects in gonadal morphology using a dissecting microscope (Figure 2A, white bars; Table S3). Temperature-sensitive alleles of *swsn-1* and *swsn-4* had a low penetrance of missing gonadal arms, as has been previously reported (Cui *et al.* 2004; Shibata *et al.* 2012). We found that *swsn-1* and *swsn-4* deletion heterozygotes were occasionally missing one of the two gonadal arms, suggesting that SWI/SNF has dose-sensitive functions in the somatic gonad. Similarly, the surviving progeny of *swsn-2.1* or *swsn-2.2* homozygotes were sometimes missing gonadal arms. Because *swsn-2.1* and *swsn-2.2* are paralogs, this may reflect a dose-sensitive requirement for *C. elegans* BAF60 in gonadogenesis. Finally, *pbrm-1* and *swsn-9* mutants had gonadogenesis defects that were more severe in the progeny of deletion homozygotes, indicating that they are required maternally and zygotically for gonadogenesis. In summary, we find that at least six SWI/SNF subunits, including the SWSN-4 ATPase, have functions in somatic gonad development.

### SWI/SNF genes act in parallel to *ehn-3*

To ask how the SWI/SNF complex interacts with *ehn-3*, we generated double mutants using a null allele of *ehn-3*. We found that alleles of *swsn-1*, *swsn-2.1*, *swsn-2.2*, *swsn-7*, *swsn-9*, and *pbrm-1* strongly enhanced the *ehn-3(rd2)* phenotype (Figure 2A, black bars; Table S3). Importantly, the penetrance and severity of the defects were greater in double homozygotes when compared to the relevant single mutants (Table S4). This is exemplified by the interaction between *ehn-3(rd2)* and *pbrm-1(ok843)*. Wild-type worms have two U-shape gonadal arms centered on the vulva (Kimble and White 1981), whereas *ehn-3(rd2)* and *pbrm-1(ok843)* single mutants were occasionally missing one of the two gonadal arms (Large and Mathies 2010) (Table S4). In contrast, *pbrm-1(ok843)*; *ehn-3(rd2)* double mutants were frequently missing one or both gonadal arms (Figure 2, B and C) and they often had severely reduced gonads (Figure 2, D and E). This severe phenotype was almost never observed in *ehn-3(rd2)* or *pbrm-1(ok843)* single mutants. We conclude that a SWI/SNF complex acts in parallel to *ehn-3* in the somatic gonad. Because both PBAF genes interacted strongly with *ehn-3*, this suggests that the complex acting in parallel to *ehn-3* is PBAF.

### Distinct functions for PBAF and BAF subunits in the somatic gonad

To clarify the roles of specific SWI/SNF subunits in somatic gonad development, we used a tissue-specific RNAi strategy to circumvent the embryonic and larval lethality associated with the loss of SWI/SNF function. This approach uses lineage-restricted promoters to drive expression of *rde-1* in *rde-1* (RNAi-deficient) mutants, thus rescuing RNAi in these lineages (Qadota *et al.* 2007). We used the *hnd-1* promoter, which is expressed broadly in the MS, C, and D lineages and later in SGPs (Mathies *et al.* 2003). These lineages produce predominantly mesodermal cell types; therefore, we refer to this treatment as mesodermal RNAi. We used *swsn-4*, *pbrm-1*, and *let-526* as representative core, PBAF, and BAF genes, respectively. All three genes produced strong RNAi phenotypes, as evidenced by nearly complete embryonic or larval lethality in a wild-type background and gonadogenesis defects in the mesodermal RNAi background (Table S5). The penetrance of gonadal defects was lower for *pbrm-1* RNAi than for *swsn-4* and *let-526* RNAi, but it was similar to *pbrm-1* deletion alleles. Qualitatively, *swsn-4* and *let-526* RNAi resulted in a centrally located patch of gonadal tissue with no arm elongation, whereas *pbrm-1* RNAi resulted in missing gonadal arms. Therefore, mesoderm-restricted RNAi reveals strong and distinct functions for specific SWI/SNF subunits.

**Figure 3:**
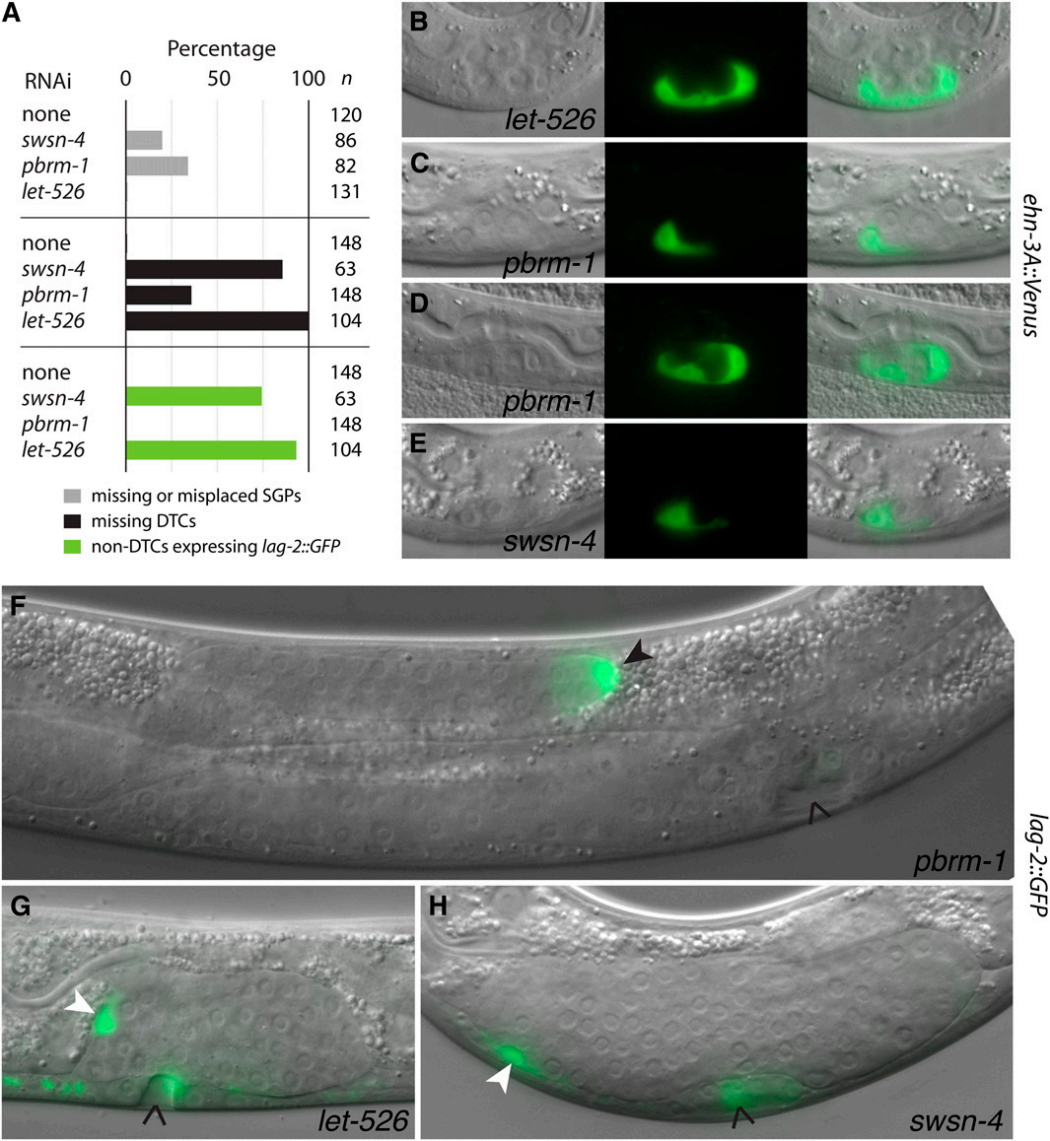
SWI/SNF subunits have distinct phenotypes in the somatic gonad. RNAi was performed in the mesodermal RNAi strain with markers for SGPs (*ehn-3A*::Venus) and DTCs (*lag-2*::*GFP*). The gene knockdown is indicated. (A) Percentage of worms (*n*) with each observed phenotype. Missing and misplaced SGPs (gray bars) were identified using *ehn-3A*::Venus; missing DTCs (black bars) and non-DTCs expressing *lag-2*::*GFP* (green bars) were identified using *lag-2*::*GFP*. Representative images are shown in (B–H). (B–E) DIC (left), fluorescence (center), and overlaid images (right) of L1 larvae expressing *ehn-3A*::Venus in SGPs. Two SGPs were present at the poles of the primordium in wild-type and *let-526* RNAi (B). SGPs were sometimes missing (C, E) or could be located more centrally within the primordium (D) in *pbrm-1* and *swsn-4* RNAi. (F–H) Overlaid fluorescence and DIC images of L4 larvae expressing *lag-2*::*GFP*. Wild-type worms express *lag-2*::*GFP* brightly in two crescent-shape DTCs located at the ends of the gonadal arms. DTCs were missing in *pbrm-1*, *swsn-4*, and *let-526* RNAi and additional gonadal cells expressing *lag-2*::*GFP* (non-DTCs) were observed in *swsn-4* and *let-526* RNAi. (F) The anterior arm was present and contained a DTC with typical morphology (black arrowhead); the posterior arm was absent (not shown). (G, H) No DTCs were present; however, *lag-2*::*GFP*–expressing cells were seen in the gonad (white arrowheads). The vulva (carat) also contained cells expressing *lag-2*::*GFP*, which were evident in longer exposures (G, H). Fluorescence exposures were 200 ms (B–E), 1 ms (F), and 10 ms (G, H).

To elucidate the developmental processes controlled by specific SWI/SNF subunits, we built molecular markers into the mesodermal RNAi strain. We used *ehn-3A*::*Venus* to mark SGPs at the L1 stage and *lag-2*::*GFP* to mark DTCs at the L4 stage (Siegfried *et al.* 2004). We began by examining L1 larvae for the presence and position of the SGPs. Wild-type worms have two SGPs located at the poles of the gonadal primordium (Kimble and Hirsh 1979). Similarly, SGPs were almost always present at the poles of the primordium in *let-526* RNAi (Figure 3B). By contrast, SGPs were sometimes absent or misplaced within the primordium in *pbrm-1* and *swsn-4* RNAi (Figure 3, C–E). Therefore, *swsn-4* and *pbrm-1*, but not *let-526*, are required for the presence and positioning of SGPs.

The hermaphrodite DTCs have at least two functions: to lead migration of the two gonadal arms and to promote mitotic proliferation in the germline (Kimble and Hirsh 1979; Kimble and White 1981). Wild-type worms have one DTC at the end of each gonadal arm. We found that *pbrm-1*, *swsn-4*, and *let-526* RNAi all resulted in missing DTCs (Figure 3A); however, they appeared to affect different developmental processes. *pbrm-1* RNAi resulted in occasional missing DTCs that correlated with missing gonadal arms. When DTCs were present, they had typical morphology and were located at the end of elongated gonadal arms (Figure 3F). Furthermore, the percentage of worms with missing SGPs was approximately the same as the percentage of worms with missing DTCs, suggesting that missing DTCs are a secondary consequence of missing SGPs. By contrast, *swsn-4* and *let-526* RNAi often resulted in unextended gonads that lacked normal DTCs but nonetheless contained cells expressing *lag-2*::*GFP* (Figure 3, G and H). These cells expressed *lag-2*::*GFP* at a lower level, they did not have the typical crescent-shape of DTCs, and they did not lead migration of the gonadal arms. Therefore, they did not appear to be normal DTCs. We conclude that *swsn-4* and *let-526* are required for DTC formation and/or function.

In summary, we found that *pbrm-1* RNAi resulted in missing and misplaced SGPs with a concomitant loss of DTCs, whereas *let-526* RNAi resulted in missing DTCs with no effect on SGPs. Furthermore, *swsn-4* RNAi resulted in missing and misplaced SGPs and a higher percentage of missing DTCs, indicating that the *swsn-4* phenotype is an aggregate of the *let-526* and *pbrm-1* phenotypes. These observations are consistent with the idea that the SWSN-4 ATPase acts with PBRM-1 to control SGP development and LET-526 to control DTC development in the somatic gonad.

### A common *hnd-1* and PBAF phenotype

**Figure 4:**
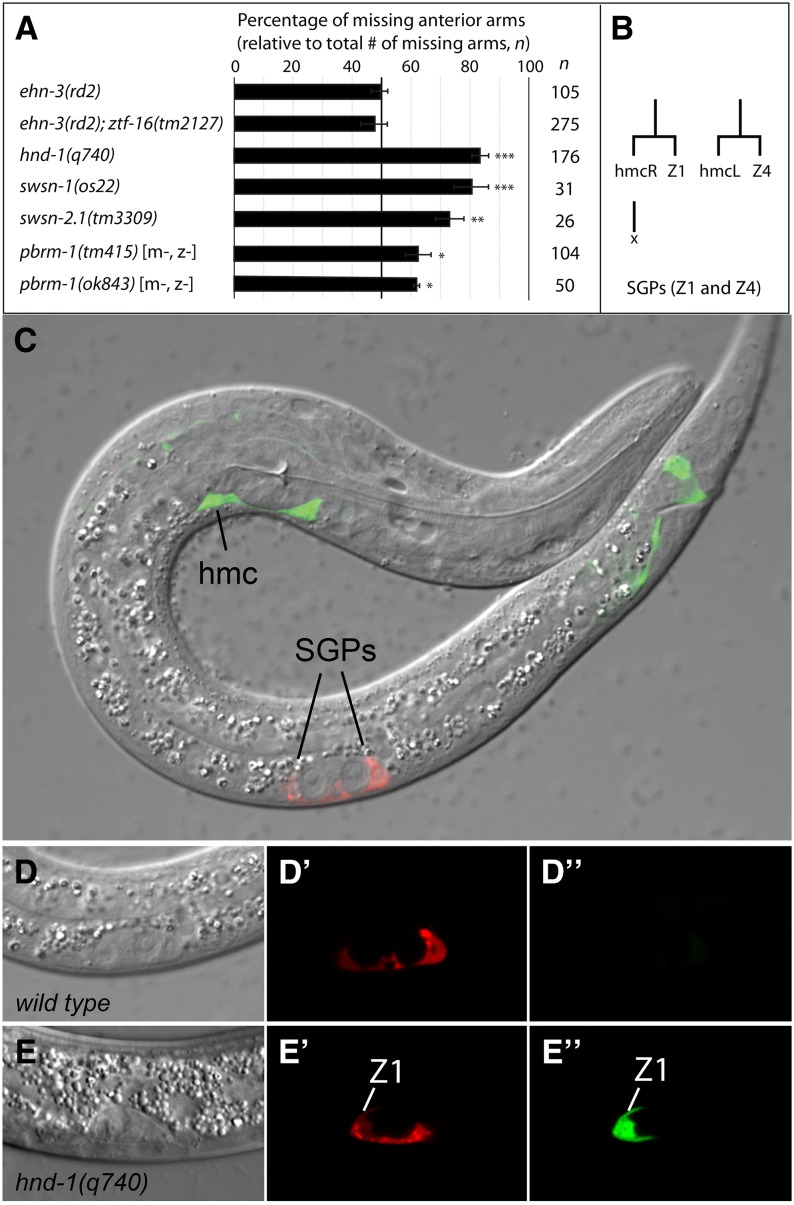
SGPs have a mixed fate in *hnd-1* mutants. (A) *hnd-1* and SWI/SNF mutants are more likely to be missing the anterior gonadal arm. The percentage of missing anterior gonadal arms (relative to the total number of missing arms, *n*) is reported. Unpaired *t*-tests were used for statistical comparisons; *ehn-3(rd2)* was compared and the significance is indicated (*P ≤ 0.05; **P ≤ 0.01; ***P ≤ 0.001). (B) SGPs (Z1/Z4) and head mesodermal cells (hmcL/R) are sisters. hmcR dies by programmed cell death (x), whereas hmcL differentiates as the single hmc. (C–E) Overlaid DIC and fluorescence images showing *ehn-3*::*tdTomato* (red) and *arg-1*::*GFP* (green) expression in L1 larvae. (D, E) Higher-magnification views of SGPs are shown with fluorescent channels displayed separately (red′, green′′). (C, D) Wild-type worms express *ehn-3*::*tdTomato* in SGPs (D′) and *arg-1*::*GFP* in the hmc and tail neurons. (E) *hnd-1* mutants often express *arg-1*::*GFP* in one or both SGPs (E′′); these SGPs also express *ehn-3*::*tdTomato* (E′). The hmc in *hnd-1* mutants only expresses *arg-1*::*GFP* (not shown).

During the course of this work, we noticed that SWI/SNF mutants exhibited two phenotypes typical of *hnd-1* mutants. First, *swsn-4* and *pbrm-1* RNAi resulted in missing or misplaced SGPs, as seen in *hnd-1* mutants (Mathies *et al.* 2003). Second, SWI/SNF and *hnd-1* mutants showed a bias toward missing anterior gonadal arms (Figure 4A). These phenotypes were not seen in other mutants affecting SGP development, such as *ehn-3(rd2)* and *ehn-3(rd2)*; *ztf-16(tm2127)*, suggesting that *hnd-1* and PBAF might control a common process.

Each gonadal arm develops from one of two SGPs that sit at opposite poles of the gonadal primordium. The anterior SGP (Z1) generates the anterior gonadal arm and the posterior SGP (Z4) generates the posterior gonadal arm (Kimble and Hirsh 1979). To ask if Z1 and Z4 are differentially affected in *hnd-1* mutants, we examined L1 larvae using *ehn-3A*::GFP to mark SGPs (Welchman *et al.* 2007). We found that *hnd-1(q740)* mutants were frequently missing one or both SGPs and the missing SGP was twice as likely to be Z1 (66.7%; *n* = 48). Therefore, differences between Z1 and Z4 are evident at the L1 stage in *hnd-1* mutants and could account for the higher percentage of missing anterior gonadal arms. The sisters of Z1 and Z4 have different fates: the sister of Z1 is hmcR, which undergoes programmed cell death, whereas the sister of Z4 is hmcL, which differentiates as the single head mesodermal cell (Figure 4B) (Sulston *et al.* 1983). We reasoned that defects in the cell fate decision between SGPs and their sisters might explain the higher percentage of missing Z1 cells in *hnd-1* mutants. To explore this, we examined *hnd-1* mutants using *ehn-3A*::*tdTomato* to mark SGPs and *arg-1*::*GFP* to mark hmcs (Kostas and Fire 2002). In wild-type L1 larvae, *arg-1*::*GFP* is expressed in the hmc and cells in the tail, whereas *ehn-3A*::*tdTomato* is expressed in the two SGPs (Figure 4, C and D). In *hnd-1* mutants, we often observed *arg-1*::*GFP* in one or both SGPs, and its expression could be very bright (Figure 4E). These SGPs also expressed *ehn-3A*::*tdTomato* and were associated with the primordial germ cells. Therefore, *hnd-1* SGPs express reporters typical of SGPs and hmcs at the L1 larval stage. Because *hnd-1* mutants always had two SGPs in embryos (Mathies *et al.* 2003), this suggests that *hnd-1* is important for SGP cell fate determination or maintenance.

**Figure 5:**
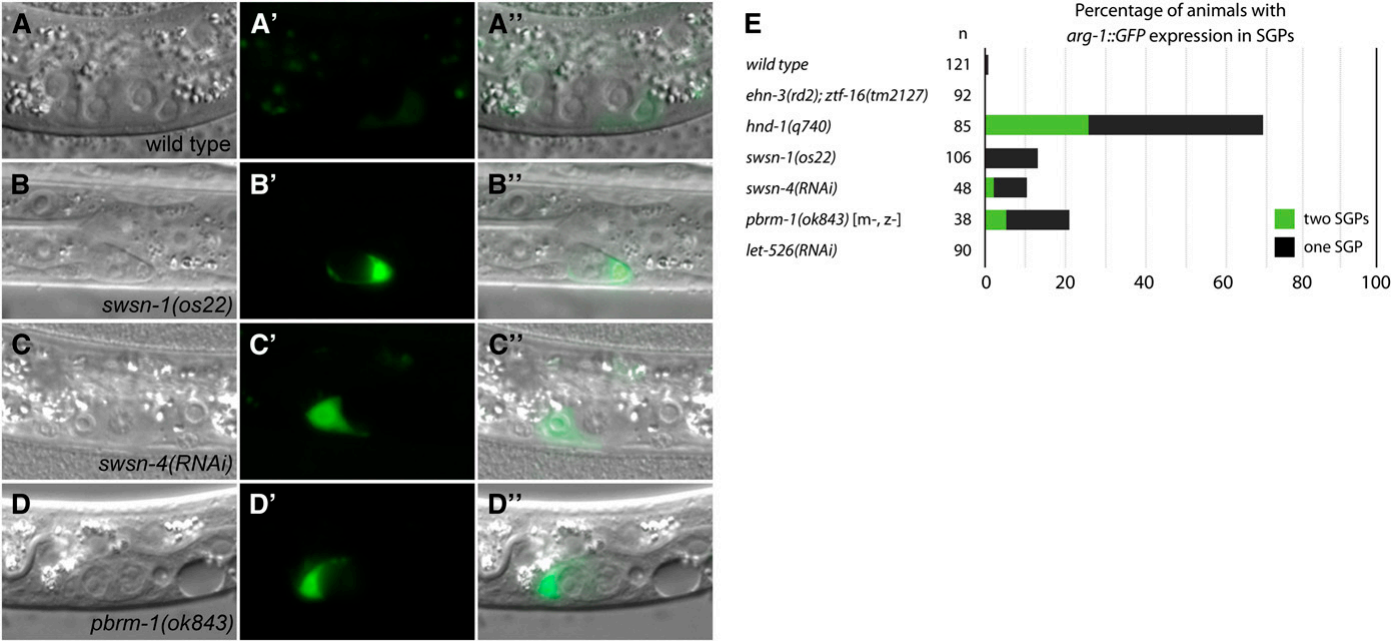
SWI/SNF mutants express *arg-1*::*GFP* in SGPs. DIC (A–D), fluorescence (A′–D′), and overlaid images (A′′–D′′) showing *arg-1*::*GFP* expression in SGPs. (A) Wild-type L1s occasionally expressed low levels of *arg-1*::*GFP* in SGPs. The exposure in (A′) is 500 ms compared to 100–200 ms in (B′–D′). (B) *swsn-1(os22)*, (C), *swsn-4(RNAi)*, and (D) *pbrm-1(ok843)* sometimes expressed *arg-1*::*GFP* in SGPs. (E) The percentage of animals with expression of *arg-1*::*GFP* in one or two SGPs is indicated. *swsn-4* and *let-526* RNAi were performed in the mesodermal RNAi strain.

We asked if SWI/SNF mutants also express *arg-1*::*GFP* in SGPs. In wild-type larvae, *arg-1*::*GFP* is expressed only rarely and at very low levels in SGPs (Figure 5A). In contrast, *swsn-1(os22)*, *swsn-4(RNAi)*, and *pbrm-1(ok843)* frequently had strong expression of *arg-1*::*GFP* in one or both SGPs (Figure 5B–E). This suggests that *hnd-1* and PBAF subunits are both important for SGP cell fate determination. Finally, *let-526(RNAi)* did not result in *arg-1*::*GFP* expression in SGPs (Figure 5E), consistent with the idea BAF and PBAF subunits have different functions in the somatic gonad.

### Relationship between SWI/SNF and *hnd-1*

We previously demonstrated that *hnd-1* acts in parallel to *ehn-3* (Mathies *et al.* 2003), and here we show that SWI/SNF genes act in parallel to *ehn-3*. Shibata *et al.* (2012) reported that *pbrm-1* mutants have reduced *hnd-1*::*GFP* expression in SGPs. Therefore, SWI/SNF may act in parallel to *ehn-3* simply by virtue of its regulation of *hnd-1*. We used genetic epistasis analysis to ask if *hnd-1* and *pbrm-1* act in a simple linear pathway. To do this, we generated *pbrm-1(tm415)*; *hnd-1(q740)* double mutants and examined them for gonadogenesis defects. The *q740* deletion is a null allele; therefore, if *pbrm-1* acts in the same pathway as *hnd-1*, then we expect the double mutant to resemble *hnd-1(q740)*. We used *pbrm-1(tm415)* for this analysis because it had only minor gonadogenesis defects when derived from a heterozygous parent (4.2 ± 1.2%; *n* = 213). We found that *pbrm-1(tm415)*; *hnd-1(q740)* double mutants had a significantly higher percentage of gonadogenesis defects (91.4 ± 6.5%; *n* = 58) when compared to *hnd-1(q740)* single mutants (50.0 ± 1.8%; *n* = 392; *P* = 0.01). We conclude that *pbrm-1* acts upstream of and in parallel to *hnd-1*.

## Discussion

In this study, we characterized alleles of 10 of the 13 *C. elegans* SWI/SNF subunits, laying the groundwork for future studies of the specific roles of individual subunits. Strong loss-of-function alleles of most SWI/SNF subunits were embryonic or larval lethal, indicating that SWI/SNF has important functions in early development. We defined different functions for SWI/SNF subunits in the somatic gonad: PBAF subunits are important for SGP development and act in parallel to *ehn-3*, whereas BAF subunits are required for DTC development. Finally, we described a common phenotype for *hnd-1* and PBAF mutants and suggest that this phenotype results from defects in the determination and/or maintenance of the SGP fate.

### PBAF subunits have unique developmental functions

We used a sensitized genetic background to examine the function(s) of SWI/SNF subunits in the somatic gonad. In total, six SWI/SNF genes interacted genetically with *ehn-3*, including alleles of core, accessory, and PBAF genes. Importantly, deletion alleles affecting homologs of two well-characterized PBAF subunits, PBRM-1/BAF180 and SWSN-7/BAF200, both strongly enhanced the *ehn-3* phenotype. This suggests that a PBAF complex acts in parallel to *ehn-3* in the somatic gonad. By contrast, SWSN-7 and PBRM-1 appear to perform different functions in early development: *swsn-7* mutants arrest at the comma stage of embryogenesis, whereas *pbrm-1* mutants arrest predominantly at the L1 larval stage. We used strong loss-of-function alleles and we compared the phenotypes resulting from loss of maternal and zygotic gene activity. Therefore, our data indicate that *swsn-7* and *pbrm-1* have similar functions in gonadogenesis and distinct functions in early development. This suggests that PBRM-1 and SWSN-7 are not dedicated subunits of the PBAF complex, but converge to form a PBAF complex during somatic gonadal development. The *Drosophila* homologs of these subunits, Bap170 and Bap180, have distinct and redundant functions in development (Carrera *et al.* 2008), and the mammalian homolog of PBRM-1, BAF180, is present in a high-molecular-weight complex that fractionates independently of PBAF (Lessard *et al.* 2007). Therefore, it is clear that PBAF subunits have additional functions that are independent of the PBAF complex. Genetic and biochemical analyses of PBAF subunits in model organisms will continue to reveal these novel functions and may also identify additional components of the PBAF complex.

### Comparison of vertebrate and invertebrate SWI/SNF complexes

Vertebrate SWI/SNF complexes are known to utilize accessory subunits encoded by duplicate genes as a mechanism to generate functional diversity (Lessard and Crabtree 2010; Wu *et al.* 2009). For example, different BAF60 proteins have been implicated in mammalian embryonic stem cell pluripotency and self-renewal, heart development, and the establishment of left–right asymmetry (Ho *et al.* 2009; Lickert *et al.* 2004; Takeuchi and Bruneau 2009; Takeuchi *et al.* 2007). The *C. elegans* genome contains two genes, *swsn-2.1* and *swsn-2.2*, encoding orthologs of the mammalian BAF60 proteins. A recent study found that *swsn-2.1*, but not *swsn-2.2*, was required for migration and serotonin production in the hermaphrodite-specific neurons (Weinberg *et al.* 2013). In contrast, we found that *swsn-2.1* and *swsn-2.2* are redundantly required for viability and they both function in parallel to *ehn-3* in the somatic gonad. The overlapping functions of *swsn-2.1* and *swsn-2.2* in embryogenesis and gonadogenesis suggest that their divergence is evolutionarily recent. Consistent with this idea, the *Drosophila* genome contains a single *Bap60* gene that is essential for viability (Moller *et al.* 2005). Because the repertoire of invertebrate accessory subunits is more limited than that of vertebrates, the utilization of BAF and PBAF subunits may play a more important role in the functional diversity of invertebrate SWI/SNF complexes.

Mammals have two homologs of SWSN-9, called BRD7 and BRD9. These proteins are found in different SWI/SNF complexes: BRD7 was purified as part of PBAF, whereas BRD9 was identified in BAF (Middeljans *et al.* 2012). Our phylogenetic analysis indicates that mammalian BRD7 and BRD9 are paralogs that resulted from a gene duplication event in the vertebrate lineage (Figure S3). We found that *swsn-9* interacted strongly with *ehn-3*, including dominant enhancement of the *ehn-3* phenotype. This is similar to PBAF genes and unlike the BAF gene *let-526*, suggesting that SWSN-9 is a PBAF subunit. We considered SWSN-9 as a potential BAF or PBAF subunit based on the mammalian biochemistry. However, our data are also consistent with the idea that SWSN-9 is a common accessory subunit and therefore is present in both BAF and PBAF complexes. In this scenario, BRD7 and BRD9 would have acquired complex specificity after the duplication of the Brd7/9 gene in vertebrates. Although we cannot distinguish between these possibilities, we favor the idea that SWSN-9 is a component of PBAF based on the striking similarity between the *swsn-9* and *pbrm-1* mutant phenotypes. An examination of other invertebrate Brd7/9 genes will shed light on the evolutionarily ancient role of this accessory subunit.

### HND-1 and PBAF subunits are important for SGP fate determination

We originally proposed that *hnd-1* was required for SGP survival, based on the following observations: *hnd-1* mutants generated two SGPs in embryos; these SGPs were sometimes absent by the L1 larval stage; and cell corpses were present and correlated with missing SGPs (Mathies *et al.* 2003). Here, we show that *hnd-1* mutants have SGPs that simultaneously express reporters typical of SGPs and their sisters, the hmcs. Based on this new evidence, we propose that *hnd-1* SGPs fail to determine or maintain their fate and, as a result, they sometimes adopt a mixed SGP/hmc fate. This, in turn, could explain the higher incidence of missing Z1 cells (the sister of Z1 dies by programmed cell death) and the higher percentage of missing anterior gonadal arms (Z1 forms the anterior gonadal arm). Furthermore, we found that PBAF mutants share this phenotype with *hnd-1* mutants, implicating SWI/SNF chromatin regulation in the molecular mechanisms that distinguish SGPs from their differentiated sisters.

The relationship between the SWI/SNF complex and HND-1 is complex. We found evidence that PBAF genes and *hnd-1* are both required for SGP cell fate determination and we observed synergistic interactions between *pbrm-1* and *hnd-1* mutants. These observations are consistent with the idea *hnd-1* and PBAF genes are partially redundant for the regulation of SGP cell fate determination genes. A variety of functional interactions between HND-1 and PBAF are possible. For example, PBAF may facilitate HND-1 binding to its target genes or, reciprocally, HND-1 may recruit PBAF to genomic regions that are important for SGP fate determination. Shibata *et al.* (2012) reported that *pbrm-1* mutants have reduced *hnd-1*::*GFP* expression in SGPs. This might reflect a loss of SGP cell fate in *pbrm-1* mutants, because *hnd-1*::*GFP* is an early marker for the SGP fate. Alternatively, *hnd-1* and *pbrm-1* may have different interactions in different cell types. *hnd-1* is expressed dynamically in mesodermal lineages and later in SGPs (Mathies *et al.* 2003), and *pbrm-1* is expressed broadly and perhaps ubiquitously (Shibata *et al.* 2012), providing ample opportunity for these genes to interact at multiple times in development. The identification of specific targets of HND-1 and PBRM-1 will allow us to explore these regulatory relationships at molecular and cellular levels.

### Relationship between SWI/SNF and *ehn-3*

We initially investigated the SWI/SNF complex as a potential partner for EHN-3. The *C. elegans* HIL gene family includes *ehn-3*, which is expressed specifically in SGPs and is important for the development of several differentiated tissues of the somatic gonad (Large and Mathies 2010). Mammalian Ikaros interacts physically with SWI/SNF (Kim *et al.* 1999; O’Neill *et al.* 2000) and is likely to control immune system development by affecting the chromatin state in hematopoietic progenitor cells (Ng *et al.* 2007). Similarly, we hypothesized that EHN-3 might establish a permissive chromatin state in SGPs via direct interactions with chromatin remodeling complexes such as SWI/SNF. However, our results clearly indicate that PBAF subunits act in parallel to *ehn-3*. It remains possible that the SWI/SNF complex interacts physically with EHN-3 and that this interaction was masked by the function of SWI/SNF with *hnd-1*. In support of this idea, we note that *ehn-3* and the BAF gene, *let-526*, are both required for the proper development of DTCs, and *let-526* did not dominantly enhance the *ehn-3* phenotype. Therefore, EHN-3 may act with BAF to promote DTC development. There are likely many multifaceted genetic and physical interactions between chromatin remodelers and cell-type–specific transcription factors. Genetic approaches will help to identify potential interaction partners, but biochemical approaches will ultimately be necessary to validate these interactions.

### Distinct roles for BAF and PBAF subunits during cell lineage progression

The BAF and PBAF complexes are biochemically defined by the presence of unique “signature” subunits (Kwon and Wagner 2007; Mohrmann *et al.* 2004). Despite sharing a common enzymatic core and several accessory subunits, BAF and PBAF subunits have distinct activities in *Drosophila* development and mammalian gene regulation (Carrera *et al.* 2008; Kaeser *et al.* 2008; Mohrmann *et al.* 2004; Moshkin *et al.* 2007; Yan *et al.* 2005). We used tissue-specific RNAi to examine the function of *C. elegans* genes encoding core (*swsn-4*), BAF (*let-526*), and PBAF (*pbrm-1*) subunits. We found that *pbrm-1* is important for SGP development, *let-526* is important for DTC development, and *swsn-4* is important for both SGP and DTC development. Our results are in agreement with a recent study characterizing *let-526* and *pbrm-1* mutants (Shibata *et al.* 2012), and they further implicate SWSN-4 as the catalytic subunit of SWI/SNF complexes in the somatic gonad. Together, these results provide strong support for the idea that distinct BAF and PBAF complexes control different processes during gonadogenesis. *Drosophila* BAP and PBAP complexes bind to different chromosomal sites and regulate distinct sets of target genes (Mohrmann *et al.* 2004; Moshkin *et al.* 2007). Similarly, we predict that *C. elegans* BAF and PBAF regulate distinct sets of target genes to control different processes in somatic gonadal development.

Recent results suggest a critical role for SWI/SNF complexes in the progression of multipotent progenitors to differentiated neurons in the mammalian nervous system. Distinct complexes are present in neural stem/progenitor cells (npBAF) and postmitotic neurons (nBAF) and result from the utilization of different paralogs of BAF45 and BAF57 (Lessard *et al.* 2007; Wu *et al.* 2007). The sequential utilization of SWI/SNF complexes may also be important in *C. elegans* hypodermal development, for which it has been suggested that the *let-7* microRNA promotes a switch between BAF and PBAF complexes concomitant with differentiation in the hypodermis (Hayes *et al.* 2011). Here, we provide evidence that PBAF subunits are required in multipotent progenitors (SGPs), whereas BAF subunits are required in a differentiated cell type of the somatic gonad (DTCs). This suggests distinct SWI/SNF chromatin remodelers are utilized over the course of cell lineage progression and underscores the importance of dynamic gene expression changes accompanying the transition from multipotency to differentiation. With the fully defined cell lineage in *C. elegans*, one day it should be possible to map these gene expression changes onto the developmental program with single cell precision.

## Supplementary Material

Supporting Information
