# Supplementary material for: Caenorhabditis elegans SWI/SNF Subunits Control Sequential Developmental Stages in the Somatic Gonad
Source: G3 (Bethesda). 2014 Jan 8;4(3):471–83. doi: 10.1534/g3.113.009852 (PMC3962486; doi:10.1534/g3.113.009852)
Supplement: Supporting Information [file supp_4_3_471__index.html]

Caenorhabditis elegans SWI/SNF Subunits Control Sequential Developmental Stages in the Somatic Gonad — Supporting Information 

# *Caenorhabditis elegans* SWI/SNF Subunits Control Sequential Developmental Stages in the Somatic Gonad

## Supporting Information for Large and Mathies, 2014

**Files in this Data Supplement:**

- Supporting Information - Figures S1-S3, Files S1-S2, and Tables S1-S5 (PDF, 885 KB)
- Figure S1 - SWI/SNF deletion alleles. (PDF, 315 KB)
- Figure S2 - Molecular analysis of *swsn-3* and *swsn-5* alleles. (PDF, 378 KB)
- Figure S3 - BRD7/9 Phylogeny (PDF, 429 KB)
- Table S1 - Molecular nature of SWI/SNF deletion alleles. (PDF, 251 KB)
- Table S2 - Primers used in this study. (PDF, 191 KB)
- Table S3 - SWI/SNF acts alone and in parallel to *ehn-3* during somatic gonad development. (PDF, 437 KB)
- Table S4 - Qualitative differences between *ehn-3* and *ehn-3; swsn* double mutants. (PDF, 429 KB)
- Table S5 - Tissue-specific RNAi of BAF and PBAF subunits. (PDF, 312 KB)
- File S1 - *C. elegans* SWI/SNF homologs and sequence alignments (.xlsx, 472 KB)
- File S2 - Positives from RNAi Screen (.xlsx, 63 KB)
