# Supplementary material for: Caenorhabditis elegans SWI/SNF Subunits Control Sequential Developmental Stages in the Somatic Gonad
Source: G3 (Bethesda). 2014 Jan 8;4(3):471–83. doi: 10.1534/g3.113.009852 (PMC3962486; doi:10.1534/g3.113.009852)
Supplement: Supporting Information [file supp_g3.113.009852_009852SI.pdf]

G3· Genes | Genomes | Genetics

Caenorhabditis elegans SWI/SNF subunits control sequential developmental stages in the somatic gonad

Edward E. Large[*] and Laura D. Mathies[* §]

[*] Department of Genetics, North Carolina State University, Raleigh, NC 27695-7614
[§] Department of Pharmacology and Toxicology, Virginia Commonwealth University, Richmond, VA 23298-0613

Corresponding author:
Laura D. Mathies
Department of Pharmacology and Toxicology
1220 East Broad St.
Richmond, VA 23298-0613
Phone: (804) 828-2069
Fax: (804) 828-4794
Email: ldmathies@vcu.edu

DOI: 10.1534/g3.113.009852

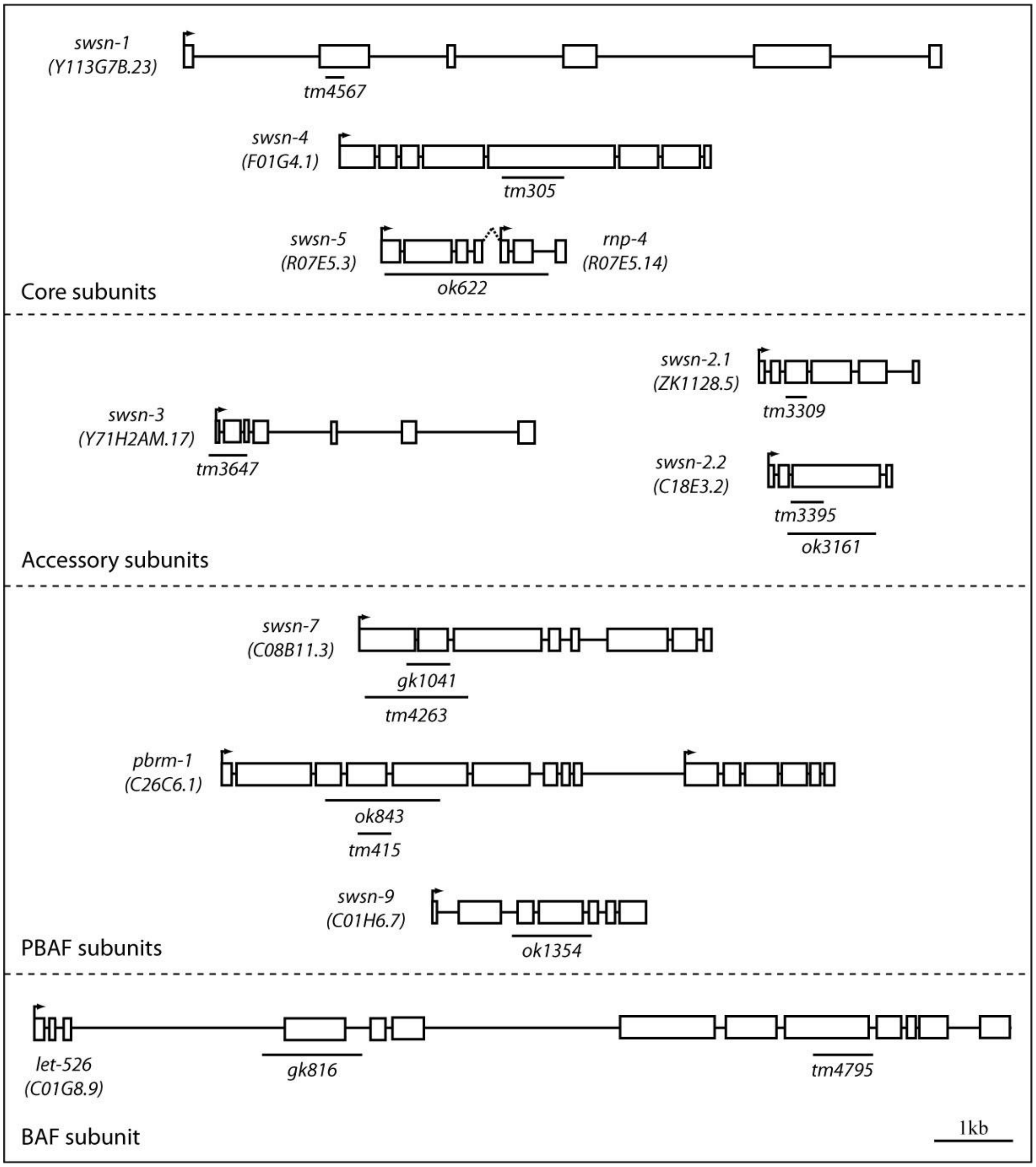

Figure S1 SWI/SNF deletion alleles. All deletion alleles were PCR amplified and verified by sequencing across the deletion breakpoint (Table S1). The extent of each deletion is indicated on the gene diagrams.

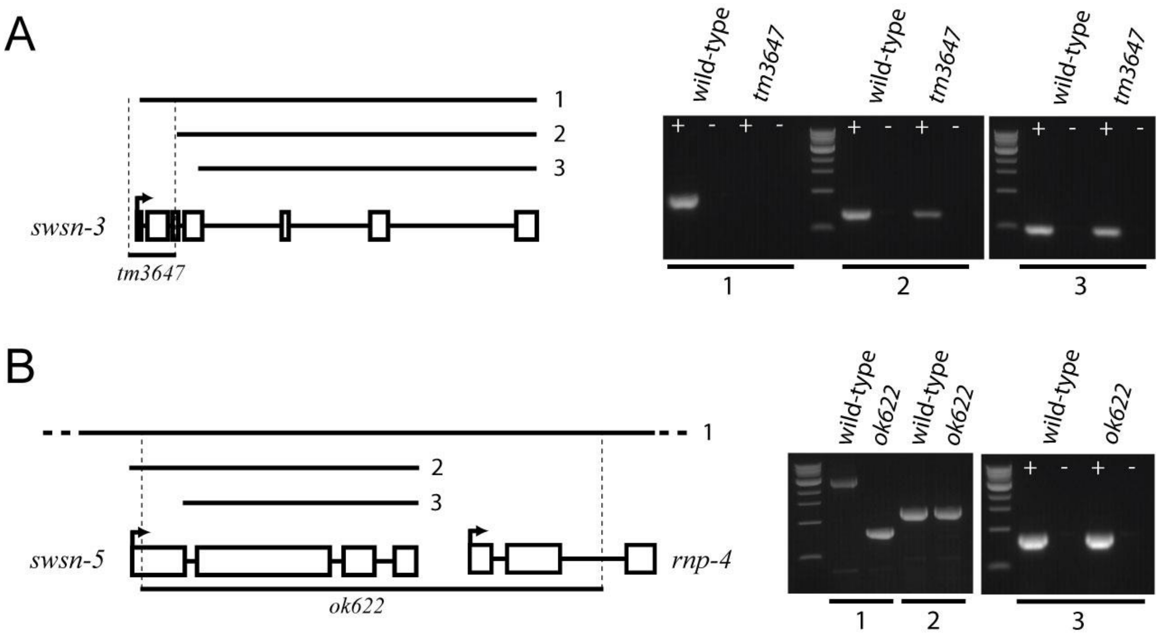

Figure S2 Molecular analysis of *swsn-3* and *swsn-5* alleles. PCR assays are indicated on gene diagrams and representative gels are shown. Template for RT-PCR was from *tm3647, ok622,* or wild-type and reactions with (+) and without (-) reverse transcriptase are indicated. Genomic DNA was from *ok622* and wild-type. Primer sequences are in Table S2. (A) RT-PCR assays were performed using primers RA561/RA562 (not shown), RA1049/RA562 (1), RA1048/RA562 (2), RA1050/RA562 (3). Transcript containing the entire *swsn-3* coding region was not detected (1), but transcripts corresponding to the non-deleted portion of the gene were detected (2, 3) in *swsn-3(tm3647)* homozygotes. (B) PCR from genomic DNA detected the *ok622* deletion (1), but it also detected the entire *swsn-5* coding region (2) and RT-PCR assays detected transcripts corresponding to the locus (2). PCR and RT-PCR assays were performed using primers RA845/846 (1), RA549/RA550 (2) and RA1051/RA550 (3).

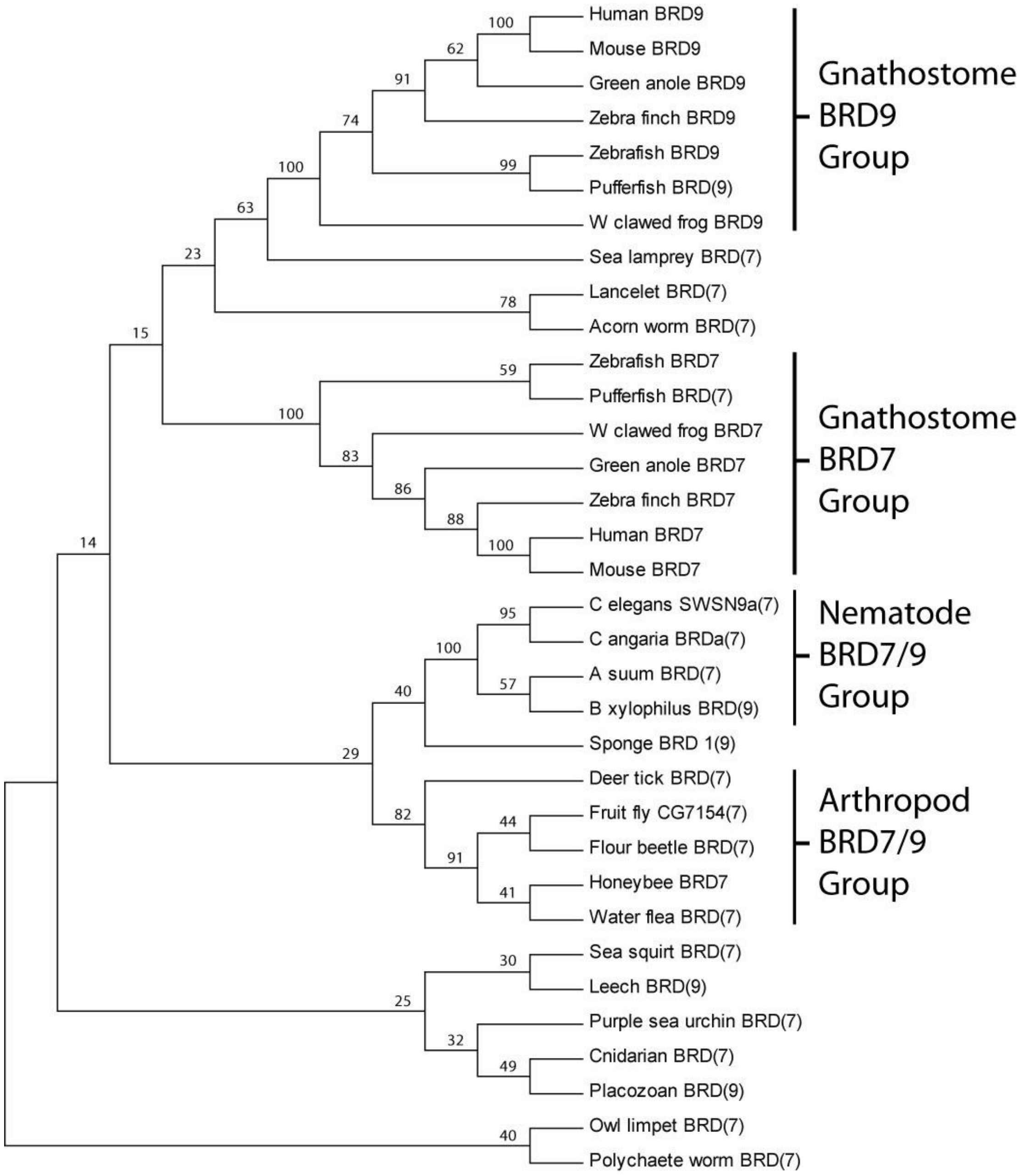

Figure S3 BRD7/9 Phylogeny. Phylogenetic tree constructed with sequences listed in File S1 using maximum parsimony; bootstrap values are indicated (*n*=100). Similar results were obtained with maximum likelihood. Sequence names indicate the sequence was more similar to human BRD7 (7) or BRD9 (9). Gnathostomes contain distinct BRD7 and BRD9 clades, while Arthropods and Nematodes contain a single BRD7/9 clade.

Files S1-S2

Available for download as Excel files at http://www.g3journal.org/lookup/suppl/doi:10.1534/g3.113.009852/-/DC1

File S1 *C. elegans* SWI/SNF homologs and sequence alignments

File S2 Positives from RNAi Screen

Table S1 Molecular nature of SWI/SNF deletion alleles

Gene	Sequence relative to wild-type
swsn-1	cactgatctcgacgaagaa --[239 BP DELETION] tctgcgacggcttgtcgcc - WT
	cactgatctcgacgaagaa TC----------------- tctgcgacggcttgtcgcc - *tm4567*
swsn-2.1	caggttcgcgaactggtt ----[241 BP DELETION] gacatattgagttaacac - WT
	caggttcgcgaactggtt TTTT----------------- gacatattgagttaacac - *tm3309*
swsn-2.2	ttcatccaaaggttttaattt [418 BP DELETION] cgcacaccacaaaccaacg - WT
	ttcatccaaaggttttaattt ----------------- cgcacaccacaaaccaacg - *tm3395*
swsn-2.2	tatgcggataaatgtattca [1122 BP DELETION] gaggaaggagttcaaaggt - WT
	tatgcggataaatgtattca ------------------ gaggaaggagttcaaaggt - *ok3161*
swsn-3	tttcatttgtttttcgta ---[432 BP DELETION] aaaaaaatcgatttaattt - WT
	tttcatttgtttttcgta AAA----------------- aaaaaaatcgatttaattt - *tm3647*
swsn-4	gaaggacaaatcagaaaagg [788 BP DELETION] acctccgtttagacggttca - WT
	gaaggacaaatcagaaaagg ----------------- acctccgtttagacggttca - *tm305*
swsn-7	aaattcatttttcaaattat [561 BP DELETION] tgcaaaatcgatttcgttcg - WT
	aaattcatttttcaaattat ----------------- tgcaaaatcgatttcgttcg - *gk1041*
swsn-7	gcaattgttgcagagttgag [1307 BP DELETION] actctccattttcaagtgt - WT
	gcaattgttgcagagttgag ------------------ actctccattttcaagtgt - *tm4263*
swsn-9	ttttcaaaaacaaattttat [1010 BP DELETION] ctcgaaaacaatgaaagaa - WT
	ttttcaaaaacaaattttat ------------------ ctcgaaaacaatgaaagaa - *ok1354*
pbrm-1	aaaaaccctgtgtcaattt [1584 BP DELETION] caaagcgaatggagtacctt - WT
	aaaaaccctgtgtcaattt ------------------ caaagcgaatggagtacctt - *ok843*
pbrm-1	gcaatgatgcaagggctt -----[427 BP DELETION] tttctcactgcaaatat - WT
	gcaatgatgcaagggctt CTCTC----------------- tttctcactgcaaatat - *tm415*
let-526	aagcatgaggttgagctcgc [760 BP DELETION] atatctttttcagagaattc – WT
	aagcatgaggttgagctcgc ----------------- atatctttttcagagaattc – *tm4795*
let-526	cacgcggagaatatataga [1268 BP DELETION] tatattatttttcgcatgac - WT
	cacgcggagaatatataga ATATA------------- tatattatttttcgcatgac - *gk816*

Table S2 Primers used in this study

Name	Sequence
RA291	GGCTCGTATGTTGTGTGGAAT
RA314	AAGGATCCTTTGTAATTTGGAAGCTGGG
RA549	caccATGTCGAGCAGCACGAAAAC
RA550	ATAGTTGAATCCACCGCCAACA
RA561	caccATGTCATCTTTCCGTCATCCGC
RA562	TTCTTCCATTTTTTCTTCCGGCT
RA714	cgactcactatagggCTGCTGACACAGTTTAATGCTCCT
RA715	cgactcactatagggCTTCTTCCATTGGGTCTTCACTATC
RA845	CTACGCGAAACGGATCAAAT
RA846	CGTGGATTGGAGAGGACAAT
RA1048	ATGATTAATAAAGGACGCGCG
RA1049	ACCGGCGAGAAATTTCCAAG
RA1050	GATACAAGAACTGTAGTTCCACACC
RA1051	GCATACTAATATCATGCTGCTGAG

Table S3 SWI/SNF acts alone and in parallel to *ehn-3* during somatic gonad development.

Genotype[a]	% Gon[b] +/- SD	*n*	
swsn-1(os22)	4.0 +/- 0.2	579	
swsn-1(ku355)	3.7 +/- 1.2	295	
swsn-1(tm4567)/rol-9(sc148)	1.5 +/- 0.9	409	
swsn-2.1(tm3309) [m-, z-]	10.7 +/- 2.1	327	
swsn-2.2(tm3395) [m-, z-]	9.0 +/- 0.5	144	
swsn-2.2(ok3161) [m+, z-]	1.2 +/- 2.3	85	
swsn-3(tm3647)	0.0 +/- 0.0	576	
swsn-4(os13)	1.7 +/- 0.7	710	
swsn-4(os13) 22.5°	3.6 +/- 3.4	140	
swsn-4(tm305)/nT1g	1.1 +/- 0.9	447	
swsn-7(gk1041) [m+, z-]	0.9 +/- 0.7	218	
swsn-9(ok1354) [m+, z-]	3.7 +/- 3.0	54	
swsn-9(ok1354) [m-, z-]	10.3 +/- 4.2	331	
pbrm-1(ok843)/hT2g	0.5 +/- 1.1	205	
pbrm-1(ok843) [m+, z-]	5.9 +/- 5.5	136	
pbrm-1(ok843) [m-, z-]	25.4 +/- 6.6	228	
pbrm-1(tm415) [m-, z-]	14.7 +/- 1.8	739	
let-526(h185)/hT2g [d]	0.0 +/- 0.0	332	
let-526(tm4795)/hT2g	0.0 +/- 0.0	162	
Genotype[a]	% Gon[b] +/- SD	*n*	p[c]
ehn-3(rd2)	22.0 +/- 2.7	441	
ehn-3(rd2); swsn-1(os22)	45.8 +/- 14.0	236	*
ehn-3(rd2); swsn-1(ku355)	54.4 +/- 3.8	406	***
swsn-2.1(tm3309) [m-, z-]*; ehn-3(rd2)*	96.9 +/- 0.5	578	***
swsn-2.2(ok3161) [m+, z-]*; ehn-3(rd2)*	57.5 +/- 4.9	113	***
swsn-3(tm3647); ehn-3(rd2)	27.0 +/- 5.8	626	NS

swsn-7(gk1041); ehn-3(rd2)	68.6 +/- 2.7	258	***
swsn-9(ok1354)/hT2g; ehn-3(rd2)	34.9 +/- 5.1	212	*
swsn-9(ok1354) [m+, z-]*; ehn-3(rd2)*	83.3 +/- 5.2	60	***
swsn-9(ok1354) [m-, z-]*; ehn-3(rd2)*	94.0 +/- 0.7	233	***
pbrm-1(ok843)/hT2g; ehn-3(rd2)	50.0 +/- 4.9	484	***
pbrm-1(ok843) [m+, z-]*; ehn-3(rd2)*	82.2 +/- 4.9	101	***
pbrm-1(tm415) [m-, z-]; *ehn-3(rd2)*	96.5 +/- 0.3	198	***
let-526(h185)/hT2g; ehn-3(rd2) [d]	22.4 +/- 7.4	361	NS
let-526(tm4795)/hT2g; ehn-3(rd2)	24.9 +/- 2.5	365	NS

[a] Maternal [m+ or m-] and zygotic [z+ or z-] contribution is indicated.

[b] Gonadogenesis defects were assessed using a dissecting microscope and the average penetrance and standard deviation (SD) are reported.

[c] Unpaired t-tests were used for statistical comparisons; *ehn-3(rd2)* was compared and the significance is indicated (NS=not significant, p≤0.05*, p≤0.01**, p≤0.001***).

[d] *let-526(h185)* is linked to *dpy-5(e61) unc-13(e450)*

Table S4 Qualitative differences between *ehn-3* and *ehn-3; swsn* double mutants

Genotype	Percentage of Animals[a] Two arms	One arm	No arms	*n*
swsn-1(os22)	96.0	4.0	0	579
swsn-1(ku355)	96.3	3.7	0	295
swsn-2.1(tm3309) [m-, z-]	89.3	7.9	2.8	327
swsn-2.2(ok3161) [m+, z-]	98.8	1.2	0	85
swsn-7(gk1041)	99.1	0.9	0	218
swsn-9(ok1354) [m-, z-]	89.7	9.4	0.9	331
pbrm-1(ok843) [m+, z-]	94.1	5.9	0	136
pbrm-1(tm415) [m-, z-]	85.3	14.1	0.7	739
ehn-3(rd2)	78.0	20.9	1.1	441
ehn-3(rd2); swsn-1(os22)	54.2	39.0	6.8	236
ehn-3(rd2); swsn-1(ku355)	45.6	34.0	20.4	406
swsn-2.1(tm3309) [m-, z-]; *ehn-3(rd2)*	3.1	10.9	86.0	578
swsn-2.2(ok3161) [m+, z-]; *ehn-3(rd2)*	42.5	43.4	13.3	113
swsn-7(gk1041); ehn-3(rd2)	31.4	47.7	20.9	258
swsn-9(ok1354) [m-, z-]; *ehn-3(rd2)*	6.0	30.1	63.9	233
pbrm-1(ok843) [m+, z-]; *ehn-3(rd2)*	17.8	55.4	26.7	101
pbrm-1(tm415) [m-, z-]; *ehn-3(rd2)*	3.5	20.2	76.3	198

[a] Gonadogenesis defects were assessed using a dissecting microscope. The percentage of animals with two gonadal arms, one gonadal arm, or no gonadal arms (including unextended and severely reduced gonads) is reported.

Table S5 Tissue-specific RNAi of BAF and PBAF subunits

RNAi	Genotype	% Gon[a] +/- SD	*n*
none	wild-type	0	>200
	hnd-1::rde-1[b]	0 +/- 0	184
swsn-4	wild-type	Emb	169
	hnd-1::rde-1[b]	99.0 +/- 1.1	99
pbrm-1	wild-type	Lvl	105
	hnd-1::rde-1[b,c]	10.2 +/- 3.3	137
let-526	wild-type	Emb	78
	hnd-1::rde-1[b]	100 +/- 0	379

[a] Gonadogenesis defects were assessed using a dissecting microscope. The average penetrance and standard deviation (SD) are reported. Embryonic (Emb) or Larval (Lvl) lethality is indicated.

[b] genotype is *unc-119(ed3); rde-1(ne219); rdIs7* [*hnd-1::rde-1*]

[c] also includes *ccIs4444* [*arg-1::GFP*]
