# Supplementary material for: Caenorhabditis elegans SWI/SNF Subunits Control Sequential Developmental Stages in the Somatic Gonad
Source: G3 (Bethesda). 2014 Jan 8;4(3):471–83. doi: 10.1534/g3.113.009852 (PMC3962486; doi:10.1534/g3.113.009852)
Supplement: Supporting Information [file supp_g3.113.009852_FigureS1.pdf]

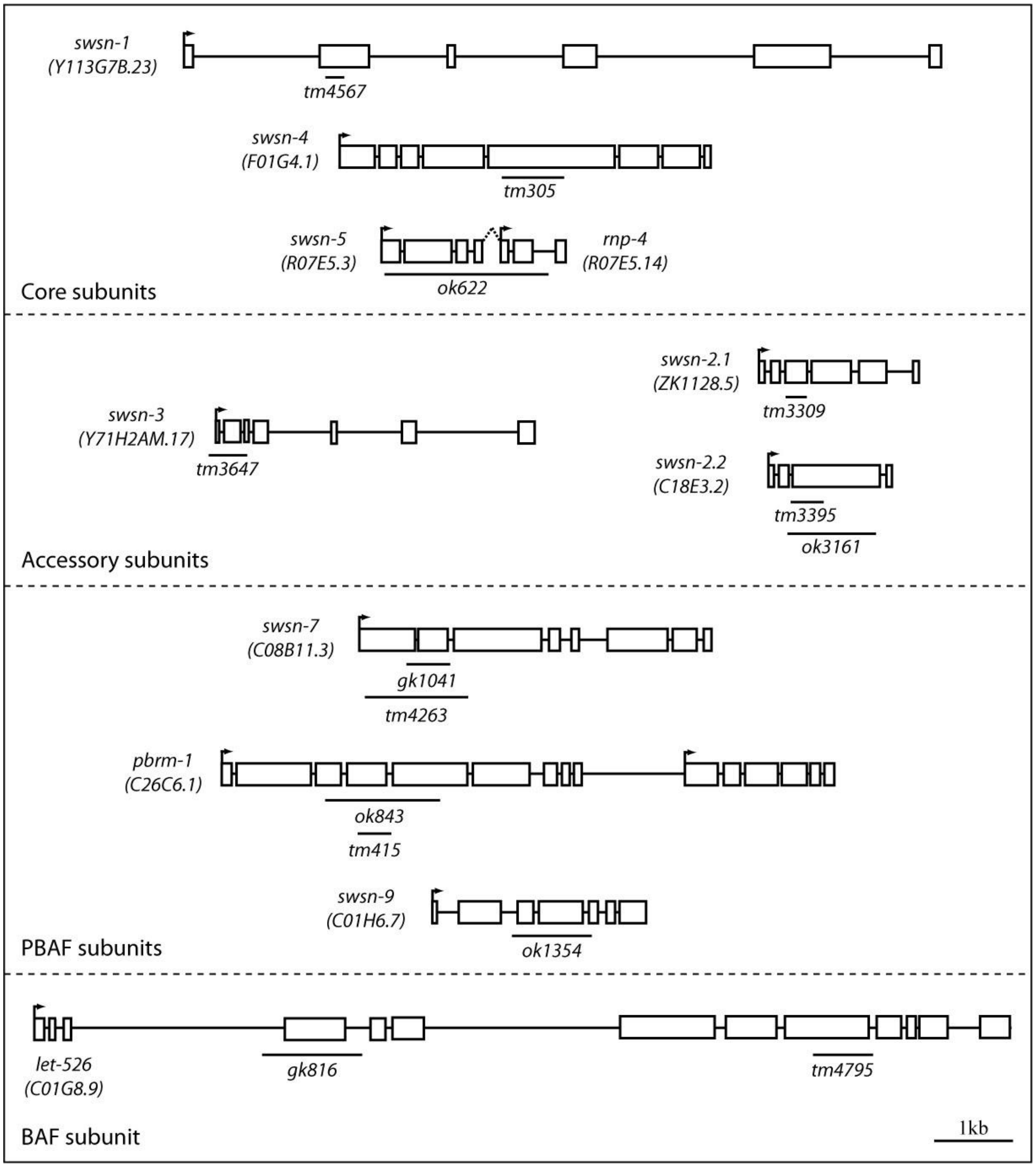

Figure S1 SWI/SNF deletion alleles. All deletion alleles were PCR amplified and verified by sequencing across the deletion breakpoint (Table S1). The extent of each deletion is indicated on the gene diagrams.
