# Supplementary material for: Caenorhabditis elegans SWI/SNF Subunits Control Sequential Developmental Stages in the Somatic Gonad
Source: G3 (Bethesda). 2014 Jan 8;4(3):471–83. doi: 10.1534/g3.113.009852 (PMC3962486; doi:10.1534/g3.113.009852)
Supplement: Supporting Information [file supp_g3.113.009852_FigureS2.pdf]

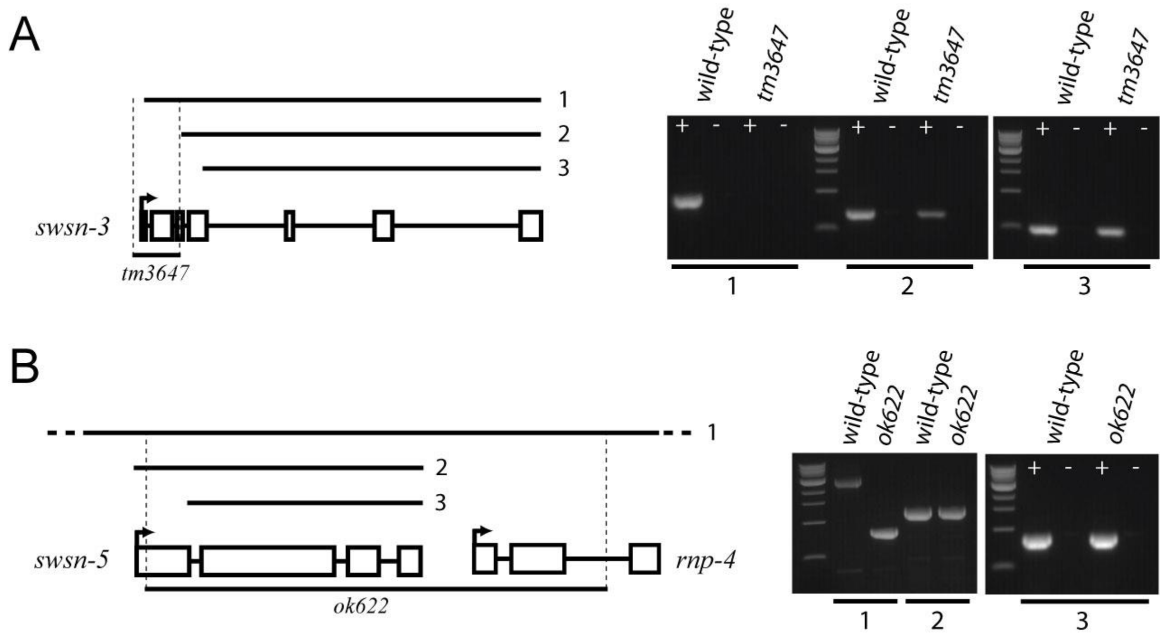

Figure S2 Molecular analysis of *swsn-3* and *swsn-5* alleles. PCR assays are indicated on gene diagrams and representative gels are shown. Template for RT-PCR was from *tm3647, ok622,* or wild-type and reactions with (+) and without (-) reverse transcriptase are indicated. Genomic DNA was from *ok622* and wild-type. Primer sequences are in Table S2. (A) RT-PCR assays were performed using primers RA561/RA562 (not shown), RA1049/RA562 (1), RA1048/RA562 (2), RA1050/RA562 (3). Transcript containing the entire *swsn-3* coding region was not detected (1), but transcripts corresponding to the non-deleted portion of the gene were detected (2, 3) in *swsn-3(tm3647)* homozygotes. (B) PCR from genomic DNA detected the *ok622* deletion (1), but it also detected the entire *swsn-5* coding region (2) and RT-PCR assays detected transcripts corresponding to the locus (2). PCR and RT-PCR assays were performed using primers RA845/846 (1), RA549/RA550 (2) and RA1051/RA550 (3).
