# Supplementary material for: Caenorhabditis elegans SWI/SNF Subunits Control Sequential Developmental Stages in the Somatic Gonad
Source: G3 (Bethesda). 2014 Jan 8;4(3):471–83. doi: 10.1534/g3.113.009852 (PMC3962486; doi:10.1534/g3.113.009852)
Supplement: Supporting Information [file supp_g3.113.009852_FigureS3.pdf]

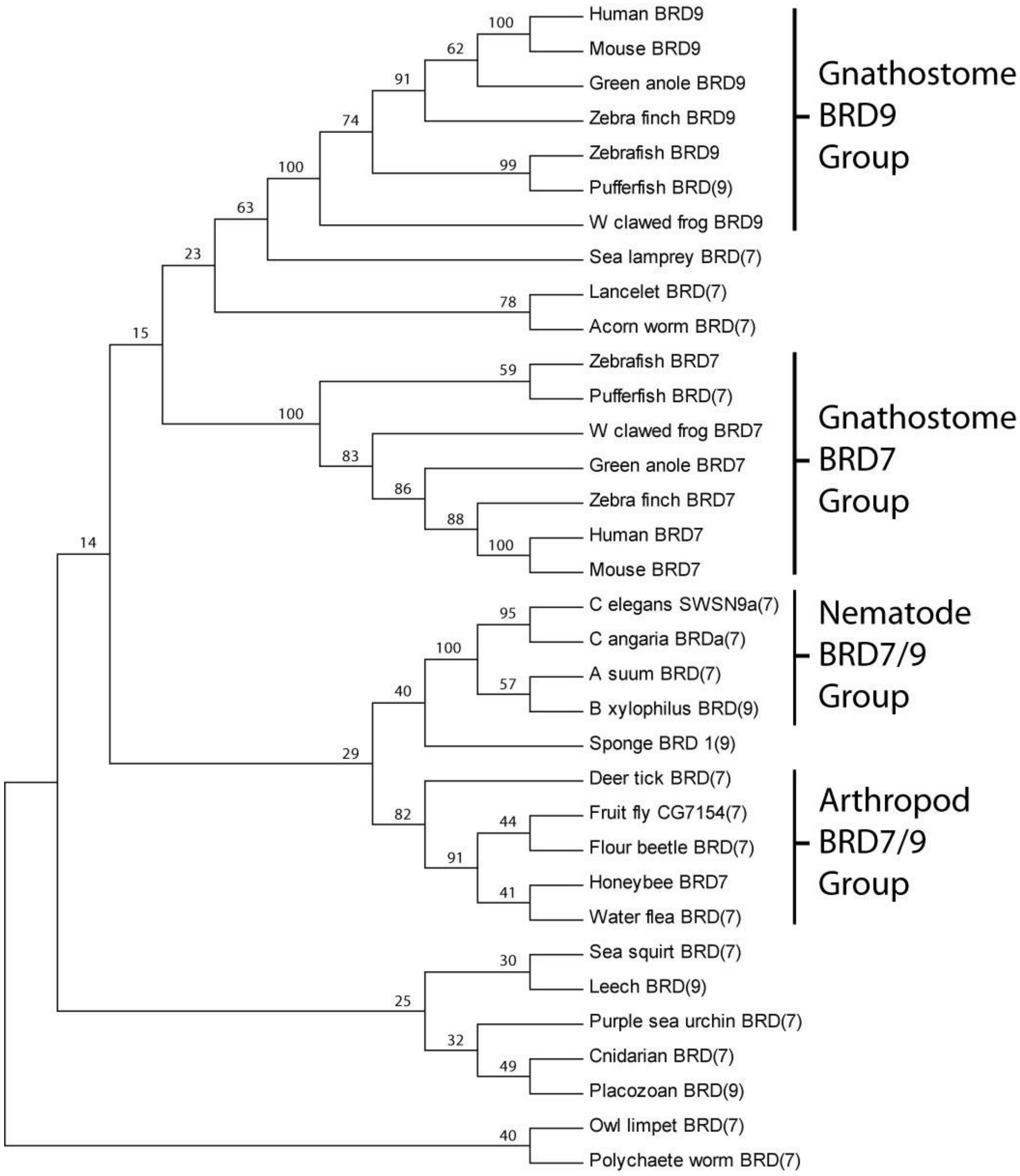

Figure S3 BRD7/9 Phylogeny. Phylogenetic tree constructed with sequences listed in File S1 using maximum parsimony; bootstrap values are indicated (*n*=100). Similar results were obtained with maximum likelihood. Sequence names indicate the sequence was more similar to human BRD7 (7) or BRD9 (9). Gnathostomes contain distinct BRD7 and BRD9 clades, while Arthropods and Nematodes contain a single BRD7/9 clade.
