# Supplementary material for: Caenorhabditis elegans SWI/SNF Subunits Control Sequential Developmental Stages in the Somatic Gonad
Source: G3 (Bethesda). 2014 Jan 8;4(3):471–83. doi: 10.1534/g3.113.009852 (PMC3962486; doi:10.1534/g3.113.009852)
Supplement: Supporting Information [file supp_g3.113.009852_TableS1.pdf]

Table S1 Molecular nature of SWI/SNF deletion alleles

Gene	Sequence relative to wild-type
swsn-1	cactgatctcgacgaagaa --[239 BP DELETION] tctgcgacggcttgtcgcc - WT
	cactgatctcgacgaagaa TC----------------- tctgcgacggcttgtcgcc - *tm4567*
swsn-2.1	caggttcgcgaactggtt ----[241 BP DELETION] gacatattgagttaacac - WT
	caggttcgcgaactggtt TTTT----------------- gacatattgagttaacac - *tm3309*
swsn-2.2	ttcatccaaaggttttaattt [418 BP DELETION] cgcacaccacaaaccaacg - WT
	ttcatccaaaggttttaattt ----------------- cgcacaccacaaaccaacg - *tm3395*
swsn-2.2	tatgcggataaatgtattca [1122 BP DELETION] gaggaaggagttcaaaggt - WT
	tatgcggataaatgtattca ------------------ gaggaaggagttcaaaggt - *ok3161*
swsn-3	tttcatttgtttttcgta ---[432 BP DELETION] aaaaaaatcgatttaattt - WT
	tttcatttgtttttcgta AAA----------------- aaaaaaatcgatttaattt - *tm3647*
swsn-4	gaaggacaaatcagaaaagg [788 BP DELETION] acctccgtttagacggttca - WT
	gaaggacaaatcagaaaagg ----------------- acctccgtttagacggttca - *tm305*
swsn-7	aaattcatttttcaaattat [561 BP DELETION] tgcaaaatcgatttcgttcg - WT
	aaattcatttttcaaattat ----------------- tgcaaaatcgatttcgttcg - *gk1041*
swsn-7	gcaattgttgcagagttgag [1307 BP DELETION] actctccattttcaagtgt - WT
	gcaattgttgcagagttgag ------------------ actctccattttcaagtgt - *tm4263*
swsn-9	ttttcaaaaacaaattttat [1010 BP DELETION] ctcgaaaacaatgaaagaa - WT
	ttttcaaaaacaaattttat ------------------ ctcgaaaacaatgaaagaa - *ok1354*
pbrm-1	aaaaaccctgtgtcaattt [1584 BP DELETION] caaagcgaatggagtacctt - WT
	aaaaaccctgtgtcaattt ------------------ caaagcgaatggagtacctt - *ok843*
pbrm-1	gcaatgatgcaagggctt -----[427 BP DELETION] tttctcactgcaaatat - WT
	gcaatgatgcaagggctt CTCTC----------------- tttctcactgcaaatat - *tm415*
let-526	aagcatgaggttgagctcgc [760 BP DELETION] atatctttttcagagaattc – WT
	aagcatgaggttgagctcgc ----------------- atatctttttcagagaattc – *tm4795*
let-526	cacgcggagaatatataga [1268 BP DELETION] tatattatttttcgcatgac - WT
	cacgcggagaatatataga ATATA------------- tatattatttttcgcatgac - *gk816*
