# Supplementary material for: Caenorhabditis elegans SWI/SNF Subunits Control Sequential Developmental Stages in the Somatic Gonad
Source: G3 (Bethesda). 2014 Jan 8;4(3):471–83. doi: 10.1534/g3.113.009852 (PMC3962486; doi:10.1534/g3.113.009852)
Supplement: Supporting Information [file supp_g3.113.009852_TableS2.pdf]

Table S2 Primers used in this study

Name	Sequence
RA291	GGCTCGTATGTTGTGTGGAAT
RA314	AAGGATCCTTTGTAATTTGGAAGCTGGG
RA549	caccATGTCGAGCAGCACGAAAAC
RA550	ATAGTTGAATCCACCGCCAACA
RA561	caccATGTCATCTTTCCGTCATCCGC
RA562	TTCTTCCATTTTTTCTTCCGGCT
RA714	cgactcactatagggCTGCTGACACAGTTTAATGCTCCT
RA715	cgactcactatagggCTTCTTCCATTGGGTCTTCACTATC
RA845	CTACGCGAAACGGATCAAAT
RA846	CGTGGATTGGAGAGGACAAT
RA1048	ATGATTAATAAAGGACGCGCG
RA1049	ACCGGCGAGAAATTTCCAAG
RA1050	GATACAAGAACTGTAGTTCCACACC
RA1051	GCATACTAATATCATGCTGCTGAG
