# Supplementary material for: Caenorhabditis elegans SWI/SNF Subunits Control Sequential Developmental Stages in the Somatic Gonad
Source: G3 (Bethesda). 2014 Jan 8;4(3):471–83. doi: 10.1534/g3.113.009852 (PMC3962486; doi:10.1534/g3.113.009852)
Supplement: Supporting Information [file supp_g3.113.009852_TableS3.pdf]

Table S3 SWI/SNF acts alone and in parallel to *ehn-3* during somatic gonad development.

Genotype[a]	% Gon[b] +/- SD	*n*	
swsn-1(os22)	4.0 +/- 0.2	579	
swsn-1(ku355)	3.7 +/- 1.2	295	
swsn-1(tm4567)/rol-9(sc148)	1.5 +/- 0.9	409	
swsn-2.1(tm3309) [m-, z-]	10.7 +/- 2.1	327	
swsn-2.2(tm3395) [m-, z-]	9.0 +/- 0.5	144	
swsn-2.2(ok3161) [m+, z-]	1.2 +/- 2.3	85	
swsn-3(tm3647)	0.0 +/- 0.0	576	
swsn-4(os13)	1.7 +/- 0.7	710	
swsn-4(os13) 22.5°	3.6 +/- 3.4	140	
swsn-4(tm305)/nT1g	1.1 +/- 0.9	447	
swsn-7(gk1041) [m+, z-]	0.9 +/- 0.7	218	
swsn-9(ok1354) [m+, z-]	3.7 +/- 3.0	54	
swsn-9(ok1354) [m-, z-]	10.3 +/- 4.2	331	
pbrm-1(ok843)/hT2g	0.5 +/- 1.1	205	
pbrm-1(ok843) [m+, z-]	5.9 +/- 5.5	136	
pbrm-1(ok843) [m-, z-]	25.4 +/- 6.6	228	
pbrm-1(tm415) [m-, z-]	14.7 +/- 1.8	739	
let-526(h185)/hT2g [d]	0.0 +/- 0.0	332	
let-526(tm4795)/hT2g	0.0 +/- 0.0	162	

Genotype[a]	% Gon[b] +/- SD	*n*	p[c]
ehn-3(rd2)	22.0 +/- 2.7	441	
ehn-3(rd2); swsn-1(os22)	45.8 +/- 14.0	236	*
ehn-3(rd2); swsn-1(ku355)	54.4 +/- 3.8	406	***
swsn-2.1(tm3309) [m-, z-]*; ehn-3(rd2)*	96.9 +/- 0.5	578	***
swsn-2.2(ok3161) [m+, z-]*; ehn-3(rd2)*	57.5 +/- 4.9	113	***
swsn-3(tm3647); ehn-3(rd2)	27.0 +/- 5.8	626	NS

swsn-7(gk1041); ehn-3(rd2)	68.6 +/- 2.7	258	***
swsn-9(ok1354)/hT2g; ehn-3(rd2)	34.9 +/- 5.1	212	*
swsn-9(ok1354) [m+, z-]*; ehn-3(rd2)*	83.3 +/- 5.2	60	***
swsn-9(ok1354) [m-, z-]*; ehn-3(rd2)*	94.0 +/- 0.7	233	***
pbrm-1(ok843)/hT2g; ehn-3(rd2)	50.0 +/- 4.9	484	***
pbrm-1(ok843) [m+, z-]*; ehn-3(rd2)*	82.2 +/- 4.9	101	***
pbrm-1(tm415) [m-, z-]; *ehn-3(rd2)*	96.5 +/- 0.3	198	***
let-526(h185)/hT2g; ehn-3(rd2) [d]	22.4 +/- 7.4	361	NS
let-526(tm4795)/hT2g; ehn-3(rd2)	24.9 +/- 2.5	365	NS

[a] Maternal [m+ or m-] and zygotic [z+ or z-] contribution is indicated.

[b] Gonadogenesis defects were assessed using a dissecting microscope and the average penetrance and standard deviation (SD) are reported.

[c] Unpaired t-tests were used for statistical comparisons; *ehn-3(rd2)* was compared and the significance is indicated (NS=not significant, p≤0.05*, p≤0.01**, p≤0.001***).

[d] *let-526(h185)* is linked to *dpy-5(e61) unc-13(e450)*
