# Supplementary material for: Caenorhabditis elegans SWI/SNF Subunits Control Sequential Developmental Stages in the Somatic Gonad
Source: G3 (Bethesda). 2014 Jan 8;4(3):471–83. doi: 10.1534/g3.113.009852 (PMC3962486; doi:10.1534/g3.113.009852)
Supplement: Supporting Information [file supp_g3.113.009852_TableS4.pdf]

Table S4 Qualitative differences between *ehn-3* and *ehn-3; swsn* double mutants

Genotype	Percentage of Animals[a]			*n*
	Two arms	One arm	No arms	
swsn-1(os22)	96.0	4.0	0	579
swsn-1(ku355)	96.3	3.7	0	295
swsn-2.1(tm3309) [m-, z-]	89.3	7.9	2.8	327
swsn-2.2(ok3161) [m+, z-]	98.8	1.2	0	85
swsn-7(gk1041)	99.1	0.9	0	218
swsn-9(ok1354) [m-, z-]	89.7	9.4	0.9	331
pbrm-1(ok843) [m+, z-]	94.1	5.9	0	136
pbrm-1(tm415) [m-, z-]	85.3	14.1	0.7	739
ehn-3(rd2)	78.0	20.9	1.1	441
ehn-3(rd2); swsn-1(os22)	54.2	39.0	6.8	236
ehn-3(rd2); swsn-1(ku355)	45.6	34.0	20.4	406
swsn-2.1(tm3309) [m-, z-]*; ehn-3(rd2)*	3.1	10.9	86.0	578
swsn-2.2(ok3161) [m+, z-]*; ehn-3(rd2)*	42.5	43.4	13.3	113
swsn-7(gk1041); ehn-3(rd2)	31.4	47.7	20.9	258
swsn-9(ok1354) [m-, z-]*; ehn-3(rd2)*	6.0	30.1	63.9	233
pbrm-1(ok843) [m+, z-]*; ehn-3(rd2)*	17.8	55.4	26.7	101
pbrm-1(tm415) [m-, z-]; *ehn-3(rd2)*	3.5	20.2	76.3	198

[a] Gonadogenesis defects were assessed using a dissecting microscope. The percentage of animals with two gonadal arms, one gonadal arm, or no gonadal arms (including unextended and severely reduced gonads) is reported.
