# Supplementary material for: Caenorhabditis elegans SWI/SNF Subunits Control Sequential Developmental Stages in the Somatic Gonad
Source: G3 (Bethesda). 2014 Jan 8;4(3):471–83. doi: 10.1534/g3.113.009852 (PMC3962486; doi:10.1534/g3.113.009852)
Supplement: Supporting Information [file supp_g3.113.009852_TableS5.pdf]

Table S5 Tissue-specific RNAi of BAF and PBAF subunits

RNAi	Genotype	% Gon[a] +/- SD	*n*
none	wild-type	0	>200
	hnd-1::rde-1[b]	0 +/- 0	184
swsn-4	wild-type	Emb	169
	hnd-1::rde-1[b]	99.0 +/- 1.1	99
pbrm-1	wild-type	Lvl	105
	hnd-1::rde-1[b,c]	10.2 +/- 3.3	137
let-526	wild-type	Emb	78
	hnd-1::rde-1[b]	100 +/- 0	379

[a] Gonadogenesis defects were assessed using a dissecting microscope. The average penetrance and standard deviation (SD) are reported. Embryonic (Emb) or Larval (Lvl) lethality is indicated.

[b] genotype is *unc-119(ed3); rde-1(ne219); rdIs7* [*hnd-1::rde-1*]

[c] also includes *ccIs4444* [*arg-1::GFP*]
